# Supplementary material for: Distinct Early Serological Signatures Track with SARS-CoV-2 Survival
Source: Immunity. 2020 Sep 15;53(3):524–532.e4. doi: 10.1016/j.immuni.2020.07.020 (PMC7392190; doi:10.1016/j.immuni.2020.07.020)
Supplement: Document S1. Figures S1–S4 [file mmc1.pdf]

Immunity, Volume *53*

Supplemental Information

Distinct Early Serological Signatures

Track with SARS-CoV-2 Survival

Caroline Atyeo, Stephanie Fischinger, Tomer Zohar, Matthew D. Slein, John Burke, Carolin Loos, Denise J. McCulloch, Kira L. Newman, Caitlin Wolf, Jingyou Yu, Kiel Shuey, Jared Feldman, Blake Marie Hauser, Tim Caradonna, Aaron G. Schmidt, Todd J. Suscovich, Caitlyn Linde, Yongfei Cai, Dan Barouch, Edward T. Ryan, Richelle C. Charles, Douglas Lauffenburger, Helen Chu, and Galit Alter

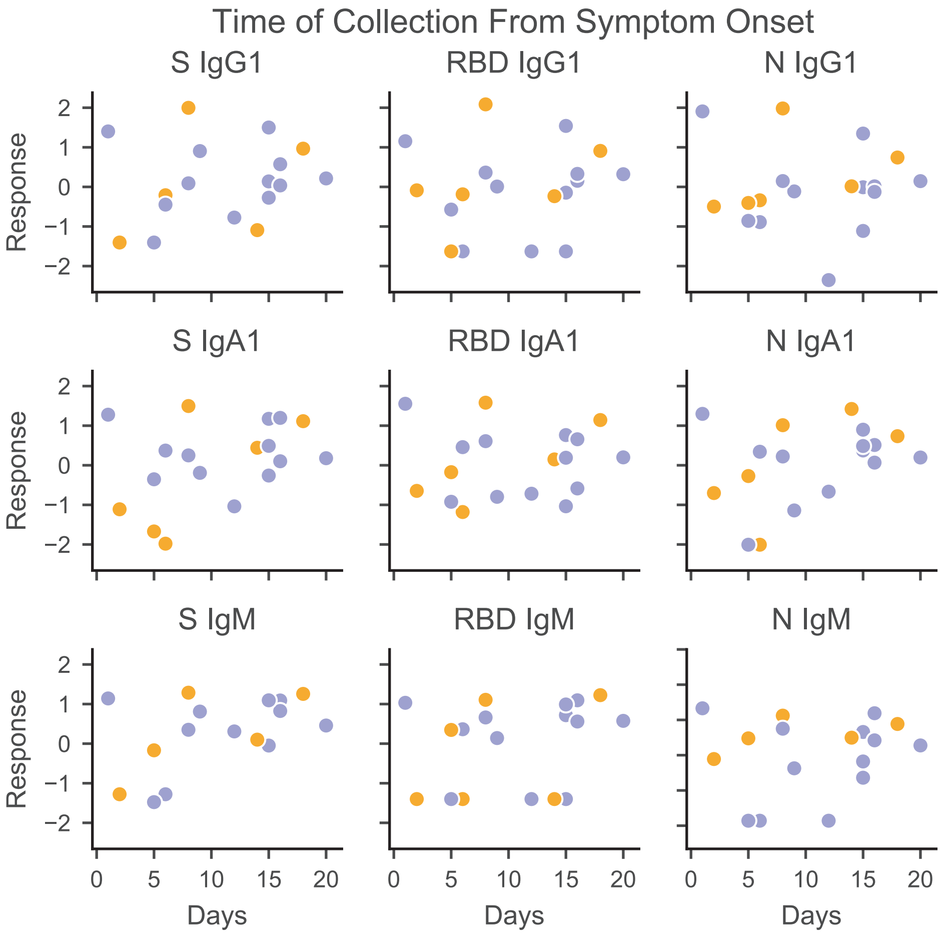

Figure S1: Individual antibody dynamics over time, related to Figure 1. The dot plots show the relationship of antibody titers against IgG1, IgA1, and IgM and the estimated time of collection from onset of symptoms across SARS-CoV-2 antigens.

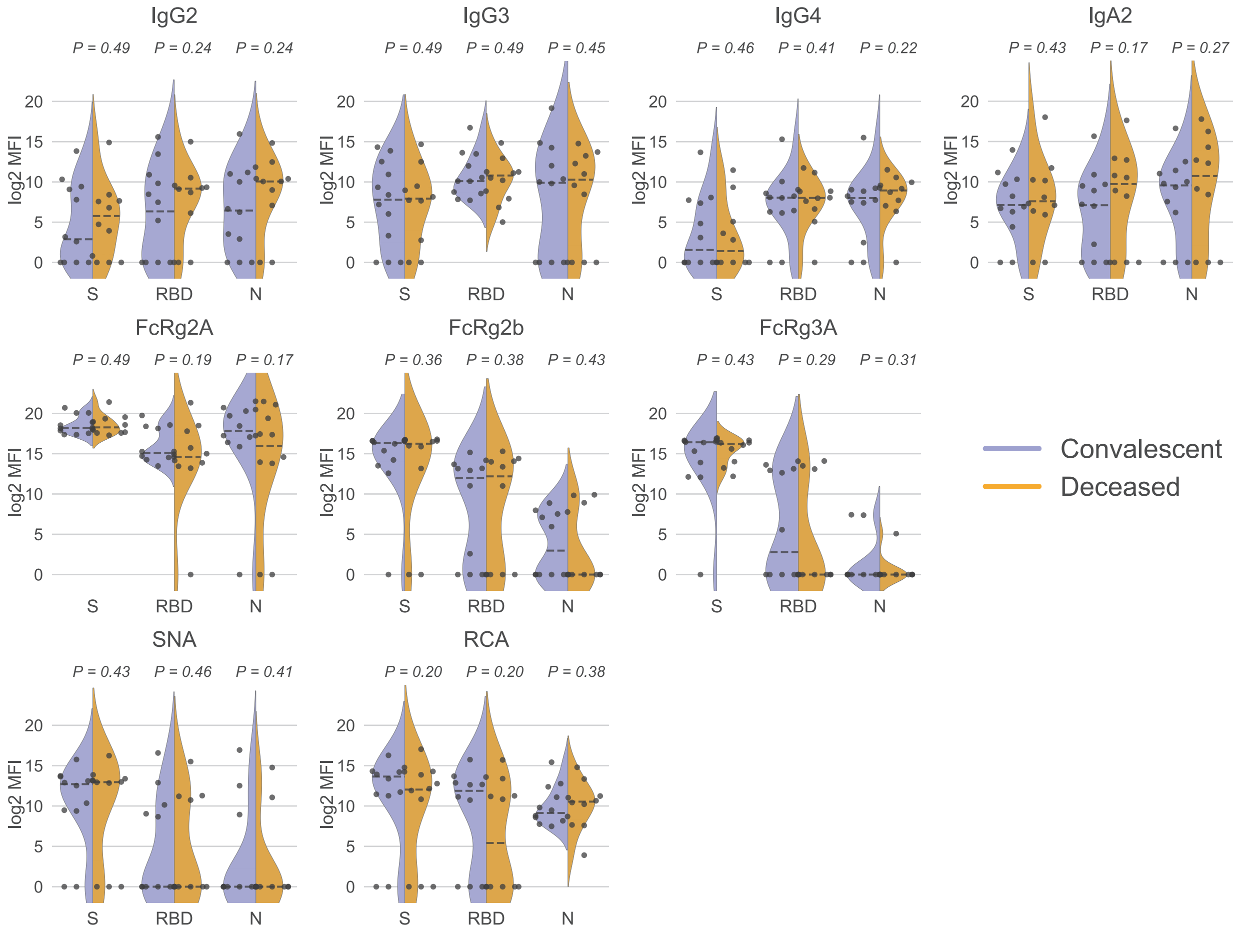

Figure S2: Individual antibody distributions, related to Figure 1. The split violin plots show the distribution of subclass, isotype, and Fc-receptor binding profiles across convalescent (purple) and deceased (orange) individuals. A Mann-Whitney U test was used to calculate p values. No significance was detected after a Holm-Bonferroni correction for multiple hypothesis testing.

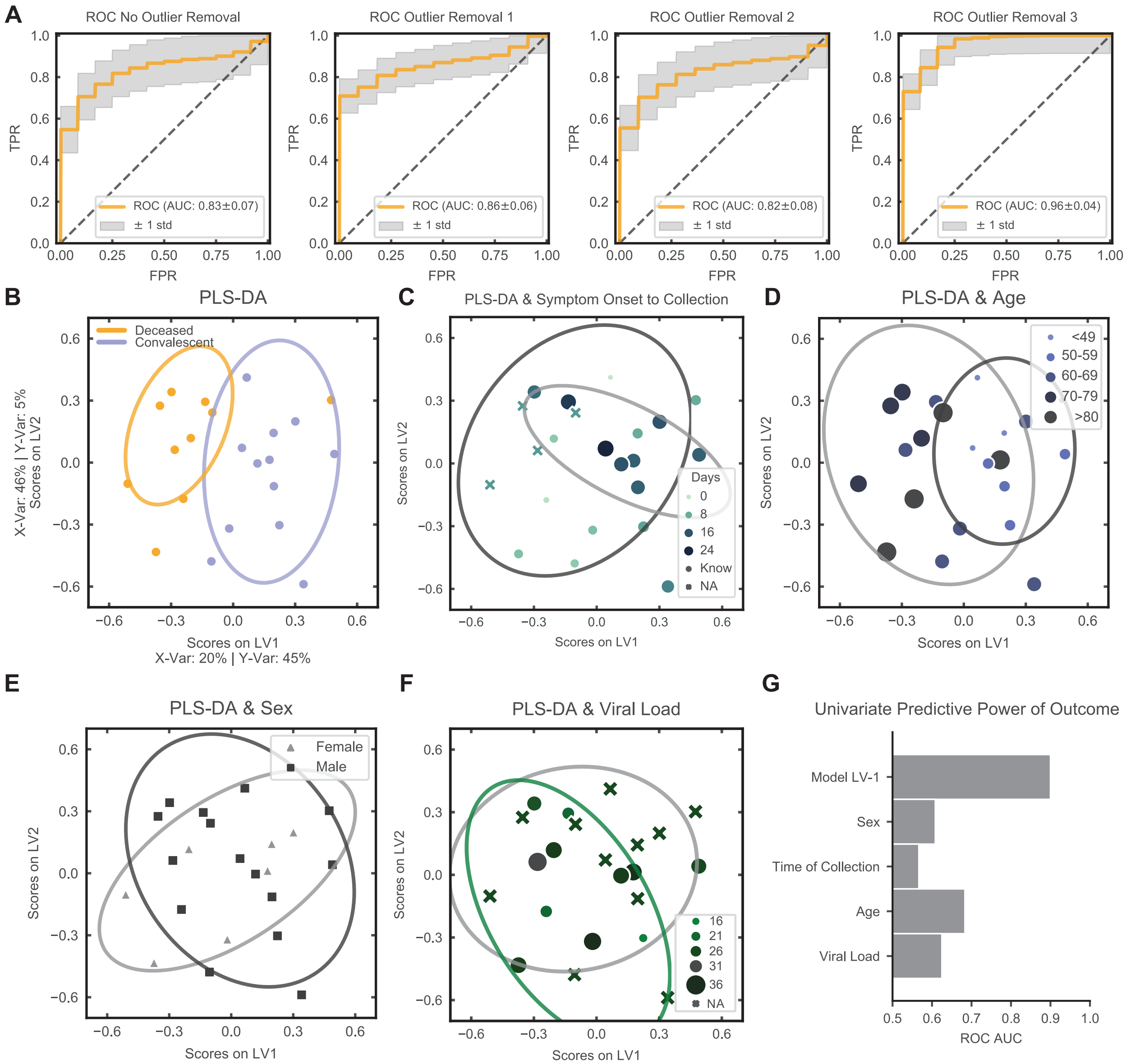

Figure S3: Sensitivity analysis, model performance, and potential influence of confounders, related to Figure 3. (**A**) Using the selected features from the original model, outliers were removed one at a time and ROC curves based on scores generated multiple times in a 5-fold cross validation framework were generated. In orange is the average performance and the grey shading represents one standard deviation. The gray dashed line represents the threshold of a random process. The AUC of the ROC curves and uncertainties are listed at the bottom of each graph. (FPR: false positive rate, TPR: true positive rate). (**B**) The original PLSDA scores plot is plotted based on the final antibody feature selection. (**C**) The same scores plot was recolored to highlight the distribution of the individuals based on days of symptom onset. The size and intensity of the dots from bright green to dark blue correspond to shorter-to-longer times, and x markers represent samples for which information was not available. (**D**) The same original scores plot was colored to reflect differences in ages across the subjects, with increased dots size and color (light blue to dark gray) showing increasing age. (**E**) The original scores plot was recolored to show the influence of sex on distributions, with males shown as dark squares and females as light gray triangles. (**F**) The original scores plot was recolored to show viral loads, with larger dots and increasing darkness (green to black) highlighting higher viral loads. (**G**) The bar graph shows the predictive power (as ROC AUC)of the original model latent variable 1 (LV1) compared to the univariate predictive power of individual demographic confounders, including sex, time to collection, age, and viral loads.

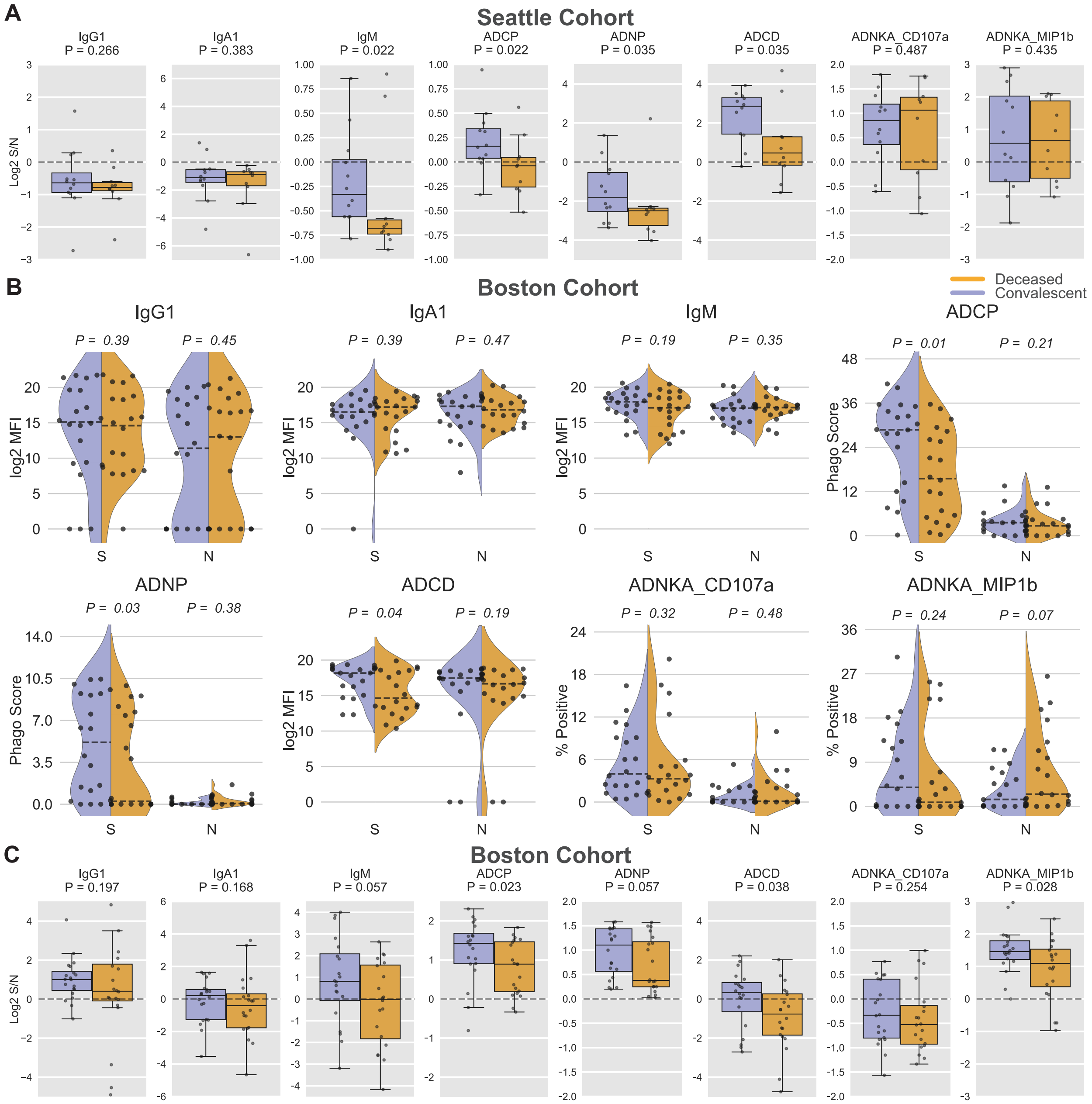

Figure S4 Univariate Spike:Nucleocapsid ratios across the Seattle/Discovery and Boston/Validation cohorts, related to Figure 4. The whisker plots show the log2 ratio of Spike to Nucleocapsid (S:N) in the (**A**) Seattle/Discovery cohort for IgG1, IgA1, IgM, antibody dependent cellular phagocytosis (ADCP), antibody dependent neutrophil phagocytosis (ADNP), antibody dependent complement deposition (ADCD), and antibody dependent NK cell activation (ADNKA) by degranulation (CD107a) and chemokine secretion (MIP1b) (from left to right). (**B**) The violin plots highlight the distributions of antibody features captured in the Boston/Validation cohort of IgG1, IgA1, IgM titers, ADCD from left to right on the top line; as well as ADCP, ADNP, and ADNKA by degranulation CD107a or MIP1b, from left to right on the bottom row. Responses are shown for Spike (S) and Nucleocapsid (N) for each read out for the convalescents (blue) and the deceased (yellow). The dashed line represents the median of the distribution. (**C**) The whisker plots show the log2 ratio of Spike to Nucleocapsid (S:N) in the Boston/Validation cohort with the same features as in (A). For both A and C the hatched line represents the point where ratios are enriched in S over N and differences were tested with a one-sided Mann-Whitney U test.
