# Supplementary material for: Distinct Early Serological Signatures Track with SARS-CoV-2 Survival
Source: Immunity. 2020 Sep 15;53(3):524–532.e4. doi: 10.1016/j.immuni.2020.07.020 (PMC7392190; doi:10.1016/j.immuni.2020.07.020)
Supplement: Document S2. Article plus Supplemental Information [file mmc3.pdf]

Report

Immunity

Distinct Early Serological Signatures Track with SARS-CoV-2 Survival

Graphical Abstract

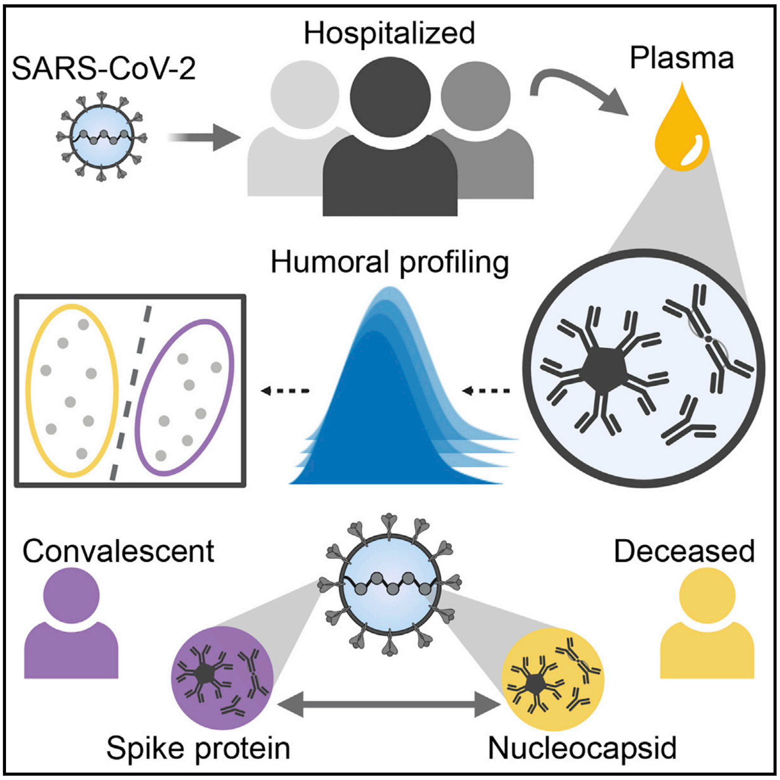

Highlights

- Limited early differences were observed in titers and neutralization across groups
- Five antibody features could collectively differentiate convalescents and deceased
- A shift in the balance of spike versus nucleocapsid immunity separated the groups
- Spike-specific phagocytic and complement fixing activity was enriched in convalescents

Authors

Caroline Atyeo, Stephanie Fischinger, Tomer Zohar, ..., Douglas Lauffenburger, Helen Chu, Galit Alter

Correspondence

helenchu@uw.edu (H.C.), galter@partners.org (G.A.)

In Brief

Although most SARS-CoV-2-infected individuals experience mild disease, a significant fraction of individuals become severely infected. Early biomarkers that predict outcome are urgently needed. Atyeo et al. demonstrate that distinct acute SARS-CoV-2 humoral immune responses exist across severely ill individuals who ultimately convalesce or pass away.

Atyeo et al., 2020, Immunity *53*, 524–532
September 15, 2020 © 2020 The Authors. Published by Elsevier Inc.
https://doi.org/10.1016/j.immuni.2020.07.020

CellPress

CellPress
OPEN ACCESS

Immunity

Report

Distinct Early Serological Signatures Track with SARS-CoV-2 Survival

Caroline Atyeo,[1,2,10] Stephanie Fischinger,[1,3,10] Tomer Zohar,[1,4,10] Matthew D. Slein,[1] John Burke,[1] Carolin Loos,[1,4] Denise J. McCulloch,[5] Kira L. Newman,[5] Caitlin Wolf,[5] Jingyou Yu,[6] Kiel Shuey,[5] Jared Feldman,[1] Blake Marie Hauser,[1] Tim Caradonna,[1] Aaron G. Schmidt,[1] Todd J. Suscovich,[8] Caitlyn Linde,[8] Yongfei Cai,[9] Dan Barouch,[6] Edward T. Ryan,[7] Richelle C. Charles,[7] Douglas Lauffenburger,[4] Helen Chu,[5,*] and Galit Alter[1,11,*]
[1]Ragon Institute of MGH, MIT, and Harvard, Cambridge, MA, USA
[2]PhD Program in Virology, Division of Medical Sciences, Harvard University, Boston, MA, USA
[3]PhD Program in Immunology and Virology, University of Duisburg-Essen, Essen, Germany
[4]Department of Biological Engineering, Massachusetts Institute of Technology, Cambridge, MA, USA
[5]Department of Medicine, University of Washington, Seattle, WA, USA
[6]Center for Virology and Vaccine Research, Beth Israel Deaconess Medical Center, Harvard Medical School, Boston, MA 02215, USA
[7]Infectious Disease Division, Massachusetts General Hospital, Boston, MA, USA
[8]SeromYx Systems, Cambridge, MA, USA
[9]Division of Molecular Medicine, Boston Children's Hospital, and Department of Pediatrics, Harvard Medical School, Boston, MA, USA
[10]These authors contributed equally
[11]Lead Contact
*Correspondence: helenchu@uw.edu (H.C.), galter@partners.org (G.A.)
https://doi.org/10.1016/j.immuni.2020.07.020

SUMMARY

As SARS-CoV-2 infections and death counts continue to rise, it remains unclear why some individuals recover from infection, whereas others rapidly progress and die. Although the immunological mechanisms that underlie different clinical trajectories remain poorly defined, pathogen-specific antibodies often point to immunological mechanisms of protection. Here, we profiled SARS-CoV-2-specific humoral responses in a cohort of 22 hospitalized individuals. Despite inter-individual heterogeneity, distinct antibody signatures resolved individuals with different outcomes. Although no differences in SARS-CoV-2-specific IgG levels were observed, spike-specific humoral responses were enriched among convalescent individuals, whereas functional antibody responses to the nucleocapsid were elevated in deceased individuals. Furthermore, this enriched immunodominant spike-specific antibody profile in convalescents was confirmed in a larger validation cohort. These results demonstrate that early antigen-specific and qualitative features of SARS-CoV-2-specific antibodies point to differences in disease trajectory, highlighting the potential importance of functional antigen-specific humoral immunity to guide patient care and vaccine development.

INTRODUCTION

SARS-CoV-2 is the newest coronavirus to cross into the human population (Wu et al., 2020b; Zhu et al., 2020). Millions of infections have been diagnosed (World Health Organization, 2020); however, the number of asymptomatic carriers is likely to far exceed these numbers (Li et al., 2020). Although the rapid spread of SARS-CoV-2, even during the asymptomatic phase of this infection, is alarming, more harrowing is our inability to predict disease trajectories among symptomatic individuals. In the absence of therapeutic agents and vaccines as countermeasures for this infection, there is an urgent need to begin to map the evolution of immunity to the pathogen to guide patient care and future immune interventions.

Although antibody responses and T cells have been linked to disease resolution (Chen et al., 2020), and neutralizing antibodies have been demonstrated to block infection in small-animal models (Quinlan et al., 2020), little is known about the antibody features that are important for protection. Neutralizing antibodies develop in the majority of SARS- and MERS-infected individuals (Chang et al., 2005; de Wit et al., 2016); however, the virus can mutate to overcome these antibody responses (He et al., 2006; ter Meulen et al., 2006). Passive immunization studies with neutralizing and poorly neutralizing antibodies have shown protection in lethal MERS infection in mice (Zhao et al., 2015, 2017), suggesting that the neutralizing and extra-neutralizing functions of antibodies may play a critical role in control and resolution of disease. Moreover, recent studies have found lower neutralization titers in younger individuals and higher neutralization among individuals with severe disease (Wu et al., 2020a; Wang et al., 2020), suggesting that antibodies may depend on additional mechanisms to clear the virus.

Antibody dynamics during the acute window of infection have been linked to differential outcomes across infections, including

 © 2020 The Authors. Published by Elsevier Inc.
This is an open access article under the CC BY license (http://creativecommons.org/licenses/by/4.0/).

CellPress
OPEN ACCESS

Table 1. Demographics of the SARS-CoV-2 Cohort from Seattle

Characteristics	Convalescent (n = 12)	Deceased (n = 10)
Female sex – no. (%)	4 (33.3)	3 (30)
Age Range – No. (%)		
Younger than 49	3 (25)	1 (10)
50-59	4 (33.3)	0 (0)
60-69	4 (33.3)	2 (20)
70-79	0 (0)	4 (40)
80 and older	1 (8.3)	3 (30)
Race or Ethnic Group – No (%)		
Asian	2 (16.7)	1 (10)
Black	0 (0)	1 (10)
White	9 (75)	7 (70)
Missing data	1 (8.3)	1 (10)
Median days from onset of symptoms to sample collection (IQR)[a]	13.5 (15–8)	7 (12–5)
Median days spent in ICU (IQR)[b]	13 (15–9)	13(14–9)
Median viral load (IQR)[c]	28.3 (30.4–26.5)	26.4 (28.375–21.725)
Interventions – No./Total No. (%)		
Chloroquines	6/12 (50)	7/10 (70)
Remdesivir	9/12 (75)	7/10 (70)
Tocilizumab	3/12 (25)	0/10 (0)
Antibiotics	8/12 (66.7)	8/10 (80)
Consequences of Disease – No./Total No. (%)		
Acute respiratory distress syndrome	5/12 (41.7)	6/10 (60)
Non-ST-elevation myocardial infarction	1/12 (41.7)	5/10 (50)

[a]IQR: interquartile range
[b]For 4 of the deceased individuals, days from symptom onset was unknown.
[c]For half of the recovered individuals, viral load measurements were not available.

HIV (Tomaras and Haynes, 2009), influenza (Cobey and Hensley, 2017), and Ebola virus infection (Saphire et al., 2018). Specifically, selection of specific antibody subclasses and functional profiles is heavily influenced by inflammatory cascades and may not only forecast disease outcomes but also point to antibody mechanisms of action vital in early pathogen control and clearance. However, whether identifiable antibody functional profiles across SARS-CoV-2 antigen specificities evolve early following infection and track differentially with disease outcome is unknown. In this study, we assembled two cross-sectional sample sets of SARS-CoV-2-infected individuals at the time of hospital admission to begin to comprehensively profile the evolution of the early SARS-CoV-2 S-specific response and to define antibody features that are predictive of disease outcome. Through this analysis, we found that deceased and convalescent individuals present different humoral profiles, with a more spike (S)-focused response in individuals who convalesced and a stronger nucleocapsid (N)-specific response in individuals who succumbed to disease.

RESULTS

Early SARS-CoV-2 Antibody Profiles in Individuals Who Ultimately Convalesce or Pass Away

Across infectious diseases, pathogen-specific antibodies can serve as biomarkers of infection and aid with early control and clearance of infection by blocking host-pathogen interactions and/or recruiting innate immune functions (Gunn and Alter, 2016). To investigate whether early SARS-CoV-2-specific humoral immune responses differ across individuals who ultimately recover or die from infection, a cohort of 22 hospitalized SARS-CoV-2-infected individuals, of whom 12 recovered and 10 died, was profiled. Samples were collected at hospital admission; all individuals were recruited within the first 20 days following symptom onset (Table 1; Figure S1) at the University of Washington, Seattle, one of the earlier epicenters in the United States (Holshue et al., 2020). Population demographics largely resemble those reported previously (Bhatraju et al., 2020), including elevated numbers of elderly men in the subset of individuals who died.

To profile the SARS-CoV-2-specific humoral immune response, we performed systems serology to determine the biophysical and functional characteristics of SARS-CoV-2-specific antibodies that recognize the SARS-CoV-2 S, the S-derived receptor-binding domain (RBD), and N. Titers of SARS-CoV-2-specific isotypes and subclasses, Fcγ-receptor binding profiles, neutralization, as well as antigen-specific innate effector functions were measured. Heterogeneous responses were observed across both populations (Figure 1A; Figure S2), and convalescents did not appear to possess quantitatively superior immune responses that could explain their different later disease course. Univariate analyses further confirmed that no significant differences were observed in SARS-CoV-2-specific immunoglobulin G1 (IgG1) or IgA1 titers across S, RBD, and N (Figures 1B and 1C; Figure S2). Conversely, subtle distribution differences were observed for SARS-CoV-2-specific IgM responses, with a slight shift toward higher S-specific IgM among survivors and a trend toward increased N-specific IgM responses among individuals who died (Figure 1C). Functional antibody profiles displayed similar distributions across the cohorts for antibody-dependent cellular phagocytosis (ADCP) (Figure 1D) and neutralization (Figure 1G). Surprisingly, RBD-specific, antibody-mediated natural killer (NK) cell degranulation (NKD) and antibody-dependent neutrophil phagocytosis (ADNP), both driven by the related Fcγ-receptors FcγR3A and FcγR3B, respectively, trended toward increases among individuals who died (Figures 1D–1F). Antibody measurements were influenced minimally by time since symptom onset (Figure S1), suggesting equivalent evolution of humoral immune responses across groups. However, no single antibody feature could discriminate between the groups.

Differences in Antibody Profile Coordination between Groups

Beyond univariate differences, emerging data point to a critical role of humoral immune response coordination as a predictor of protection in some infections (Ackerman et al., 2018; Barouch

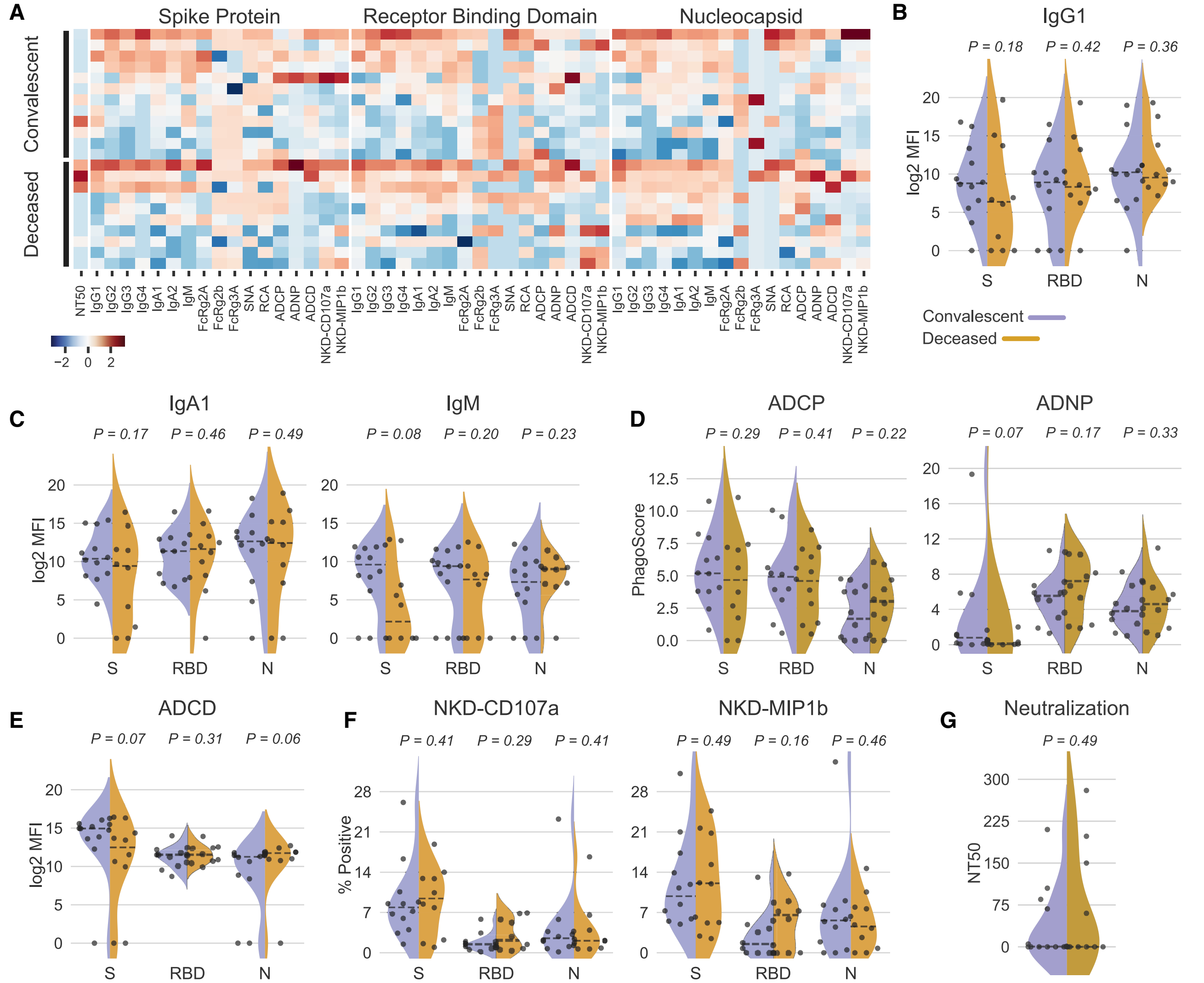

Figure 1. Heterogeneity in Antibody Responses across SARS-CoV-2 Antigens in Individuals Who Recover or Pass Away

22 plasma samples from SARS-CoV-2 infected individuals were profiled at the time of hospitalization against SARS-CoV-2 S, RBD, and N antigens.

(A) The heatmap shows the humoral immune responses across individuals who later passed away (deceased) or recovered (convalescent). The heatmap is split by SARS-CoV-2 S, RBD, and N antigens. Rows correspond to individuals. Columns correspond to antibody features (background subtracted and *Z*-scored), including neutralization, isotype, subclass, and antibody effector functions. High responses are shown in red, and low responses are depicted in blue.

(B–G): Violin plots show the distribution of each antibody feature split across convalescent (purple) and deceased (orange) individuals across antigens. The dashed gray line indicates the median value of each distribution. A two-sided Mann-Whitney *U* test was used to calculate uncorrected p values. No significance was detected after a Holm-Bonferroni correction for multiple hypothesis testing.

et al., 2015). Given the polyclonal nature of the early humoral immune response, multiple functions or features may simultaneously contribute to differential control and clearance of infection. Correlation matrices split by group were used to examine the relationships between antibody isotypes or subclasses and antibody-dependent effector functions across the groups (Figure 2A). Within both groups, isotypes and subclasses were highly correlated. Conversely, the relationship between isotype or subclass and functions differed across the two populations. Stronger correlations between titers and functions were observed in convalescent individuals (Figure 2A). Disparities were observed in NK cell and neutralizing antibody coordination between the two groups. Although not significant, individuals who died exhibited correlated isotype or subclass responses with monocyte and neutrophil phagocytosis but negative and generally poorer correlations of NK cell-activating and complement-recruiting antibody responses with all other functions(Figure 2A), suggesting that individuals who pass away develop a functionally biased humoral immune response. Although IgG1 responses were associated with all functions across the individuals who later died, diversified isotype and subclass responses were largely inversely correlated with antibody-dependent complement deposition (ADCD) and NK cell functions. This observation suggests that these individuals leverage isotype and subclass

CellPress
OPEN ACCESS

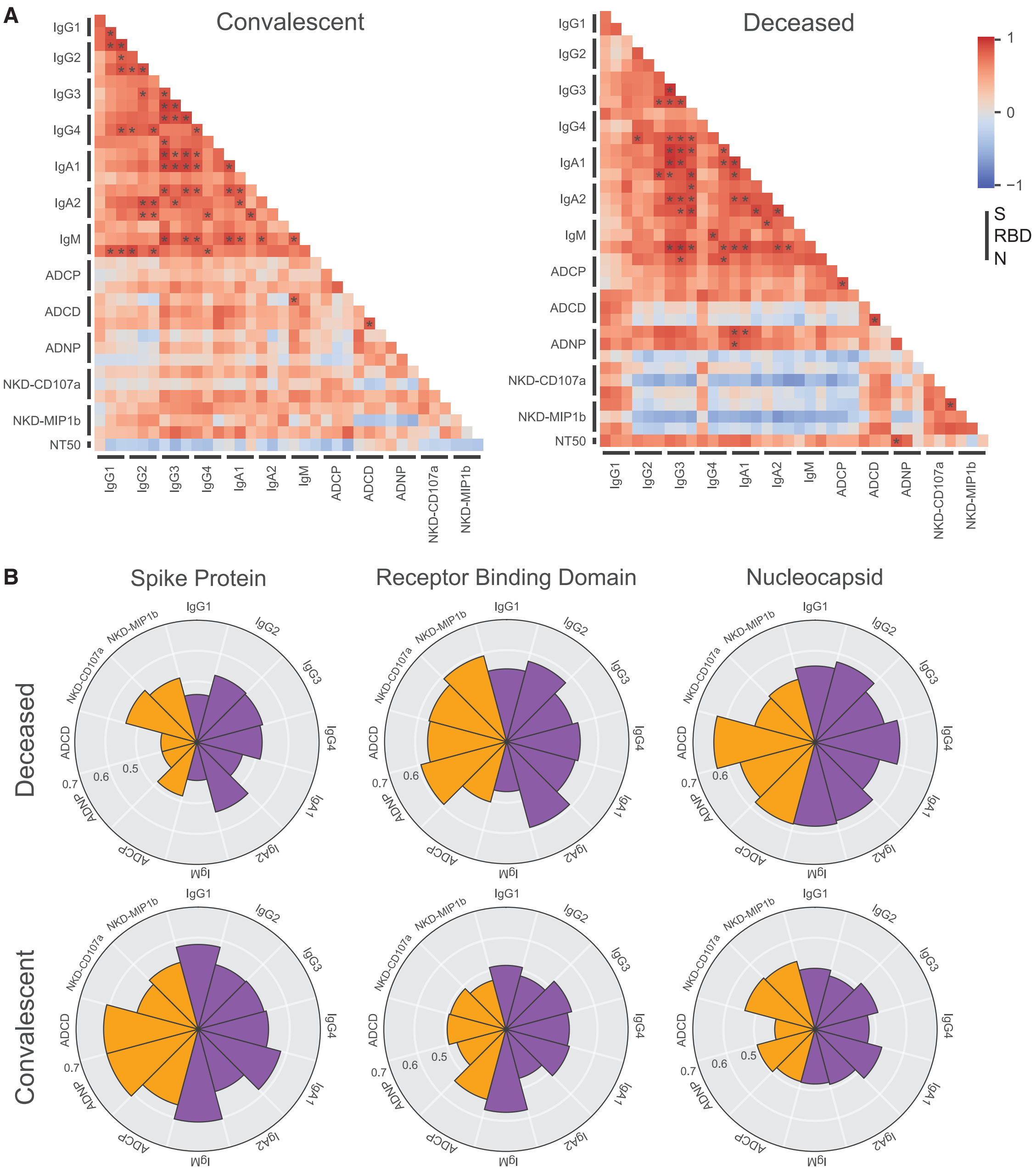

Figure 2. Deceased Individuals Showed Less Coordinated and N-Directed Antibody Responses

(A) The correlation heatmap shows pairwise Spearman correlation matrices of antigen-specific antibody titers and effector functions for convalescent (left) and deceased (right) patients. For each feature analyzed, the bar covers the S, RBD, and N antigens, shown in the legend on the right. Statistical significance is indicated by gray asterisks with Holm-Bonferroni correction for multiple hypothesis testing ($p < 0.001$). Negative correlations are indicated in blue, and positive correlations are denoted in red.

(B) The Nightingale rose plots show the mean percentile of antibody features within the deceased (top) and convalescent (bottom) groups. Plots represent the S-, RBD-, and N-specific responses across deceased (top) and convalescent (bottom) individuals. Each wedge represents a SARS-CoV-2 antibody feature. The size of the wedge depicts the magnitude of the value. The colors represent the type of feature: orange, antibody functions; purple, antibody isotypes and subclasses.

diversification in a manner that may preclude full deployment of the humoral immune response.

Conversely, convalescents overall displayed a more uniform correlation profile across subclass and isotype responses and antibody effector function. However, although neutralizing antibody responses were co-induced with isotype and subclass and effector functions among individuals who died, neutralizing antibody responses were largely inversely correlated with all antibody responses among individuals who recovered, suggesting a divergent evolution of the antigen-binding and constant domain of the antibody across these populations. These data highlight multiple early functional

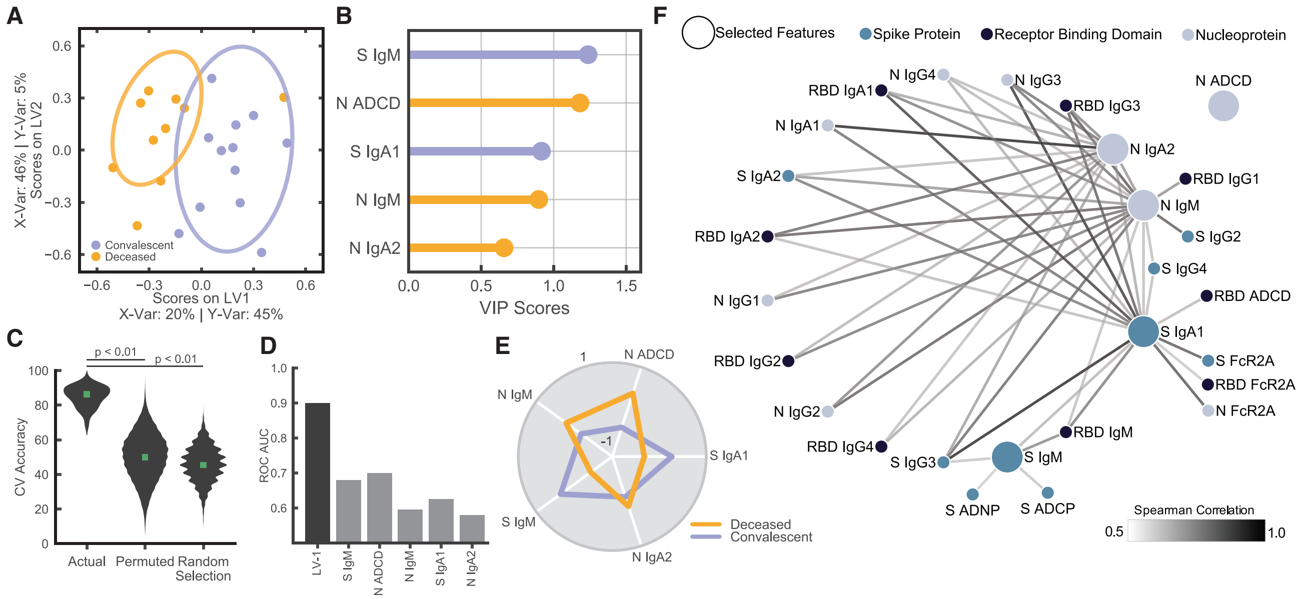

Figure 3. Select Antibody Features Distinguish Convalescent and Deceased Individuals

(A) The PLSDA score plot shows the degree of discrimination that was achievable across the groups following feature-down selection. Each dot represents an individual: convalescent (purple) and deceased (orange). Ellipses correspond to the 95% confidence intervals for each group.

(B) The line graph shows the variable importance in projection (VIP) score of the selected features. As few as 5 features were required to separate the groups. The magnitude indicates the importance of the feature in driving separation in the model. The color of the feature corresponds to the group in which the feature is enriched.

(C) The violin plots show the distributions of repeated classification accuracy tests using the actual data, shuffled labels, and randomly selected size-matched features, illustrating the performance and robustness of the model. Green squares indicate the median accuracies.

(D) The predictive power of the model built on the selected features is shown in the LV1 column. In addition, the predictive power of each individual selected feature is represented in gray. The predictive power is illustrated as the area under the curve (AUC) of the receiver operating characteristic (ROC) curves for the model (LV1) or each feature alone.

(E) The radar plot shows the *Z*-scored univariate values of the selected features across both groups.

(F) The correlation network illustrates the co-correlated features (small nodes) that are significantly correlated with the model-selected features (large nodes). Edge transparency corresponds to correlation strength. Antigens are indicated by different colors (S, teal; N, gray; RBD, black).

differences in SARS-CoV-2-specific humoral immunity between the groups.

To further probe the overall humoral profile between groups, the mean percentile of each antibody metric was determined across SARS-CoV-2 antigen specificities for both populations (Figure 2B). Nightingale rose plots reveal that deceased individuals exhibited a more N-focused humoral immune response compared with the S-centric response elicited among convalescents. In particular, higher S-specific ADCD, ADNP, and ADCP and enhanced IgG1, IgA1, and IgM responses were observed among survivors. In contrast, S-specific NK cell-activating responses were enriched in the deceased. Unexpectedly, RBD-specific responses were largely enriched among individuals who passed away, with the exception of RBD-specific monocyte phagocytosis, which was enriched among individuals who survived. These data point to antigen-specific and antibody-effector differences early in infection that differ by clinical trajectory.

Defining Signatures that Differentiate Disease Trajectory

Given the unique correlation and immunodominance profiles across the groups (Figures 2A and 2B), we next aimed to define whether a minimal set of features could be identified that could segregate individuals with different clinical outcomes. To this end, feature down-selection was performed to avoid overfitting, followed by partial least-squares discriminant analysis (PLSDA) to visualize differences (Lau et al., 2011). Despite the small numbers, separation was observed across the groups (Figure 3A). All antibody features as well as sex and interventions (Table 1) were included in the analysis, and as few as 5 features were sufficient to drive separation across the subjects (Figures 3A and 3B). S-specific IgM and IgA1 responses were enriched in survivors, whereas N-specific complement activity (ADCD), IgM, and IgA1 titers were enriched in individuals who died. These data likely relate to the immunodominant shift toward S in convalescent individuals and toward N in deceased individuals (Figure 2B). Model performance was evaluated using leave-one-out cross-validation to test the significance of the model using different sets of subjects and to test outlier effects. The model clearly outperformed (Cliff's Δ) permuted and size-matched random controls (Figure 3C). Moreover, sensitivity analysis, evaluating model performance with removal of individual outliers, highlighted the minimal effect of any given individual (Figure S3A). Furthermore, individual model features only possessed modest predictive power in resolving the groups, but collectively, combining all 5 features in latent variable 1 (LV1) exhibited improved predictive accuracy (Figure 3D). Confounding

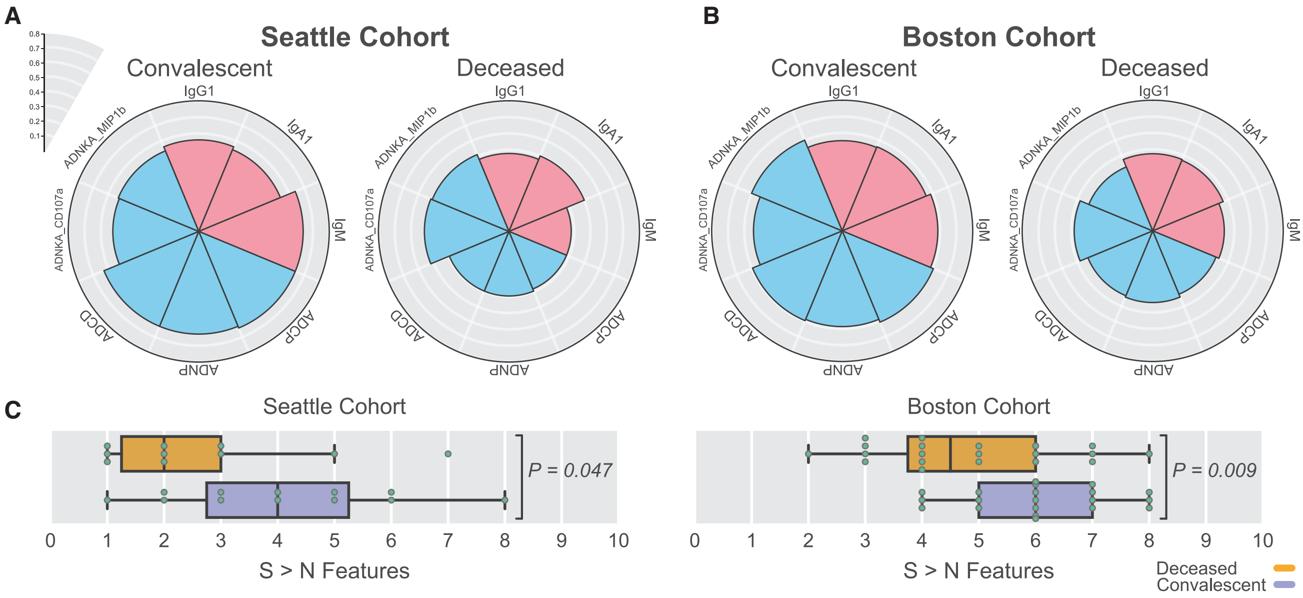

Figure 4. Converging Shift in Immunity across a Second Acute Infection Cohort

(A and B) The Nightingale rose plots show the mean percentile of the spike:nucleocapsid (S:N) ratio of each readout are depicted for (A) the Seattle or discovery cohort and (B) the Boston or validation cohort for convalescents (left) and deceased (right). Titers are shown as pink wedges and functions as blue wedges.

(C) The whisker boxplots show the number of S features that are greater than their N counterparts for all individuals in the Seattle or discovery cohort (left) and the Boston or validation cohort (right). Differences across the 2 groups were assessed using a one-sided Mann-Whitney *U* test.

features, such as days since symptom onset, sex, age, and viral load, were also overlayed on the PLSDA score plot (Figures S3B–S3F), highlighting the limited capacity of any of these features to distinguish individuals who convalesced or died. Furthermore, at individual levels, these demographic factors were poorly predictive of disease outcome, underperforming classification compared with the LV1 classification model (Figure S3G). Thus, a minimal set of SARS-CoV-2 humoral profiles, rather than demographic information, appears to significantly resolve individuals who die from those who recover.

Given that the feature down-selection algorithm selects a minimal set of features to avoid overfitting, a co-correlates network was used to explore additional features that may distinguish these two groups (Figure 3F). A larger set of co-correlates can help provide mechanistic clues related to the immunologic mechanisms by which antibodies contribute to control and clearance of infection. Thus, a co-correlate network was built, highlighting the relationship of model-selected features (large nodes) with additional highly correlated features (smaller nodes). Features enriched among individuals who later died included N-specific IgM and IgA2, which were linked to a large number of additional N- and RBD-specific poorly functional antibody features. For example, correlates of risk were linked to induction of less functional IgG subclasses, IgG2 and IgG4, pointing to early rise of dysregulated or less functional humoral immune responses as biomarkers or even drivers of ineffective control or clearance of infection. Conversely, S-specific IgM titers, enriched in convalescent individuals, were correlated with functional S-specific IgG3 responses, RBD-specific IgM, and S-specific monocyte and neutrophil phagocytosis. Moreover, S-specific IgA1 responses, also enriched among convalescents, were linked to RBD-specific complement activation (ADCD) and S-, RBD-, and N-specific FcγR2A binding, the Fcγ receptor involved in phagocytosis. Given our emerging appreciation of the role of complement and phagocytosis in vaccine-mediated protection against SARS-CoV-2 (Yasui et al., 2014), these data potentially argue for a similar role of these functions in natural protection against disease. Moreover, the data also highlight the potential importance of a less N-focused but more functional S-specific phagocytic response as an early correlate of recovery from infection.

Validation of the Skewed S-Specific Response in Convalescents

Collectively, the data point to a shift in immunodominance of S versus N functional antibody responses. To test this hypothesis, we next compared the overall ratio of S:N-specific antibody isotypes, subclasses, and functions across the groups (Figure 4A; Figure S4A). As expected, several antibody features were selectively biased toward S immunity in convalescents compared with individuals who later died, including IgM, ADCP, ADNP, and ADCD. Whether these effects were exclusive to this group of individuals from Seattle or could be generalized was next addressed in a second, larger cohort of acutely infected individuals from Boston, of whom 20 individuals convalesced and 20 died. Similar to the Seattle cohort, the Boston samples were profiled in the first 20 days following symptom onset (Table 2). Similar to the Seattle discovery cohort, although differences were observed in S- and N-specific immune responses at a univariate level, none passed multiple hypothesis correction (Figure S4B). However, when S:N ratios were compared across features, convalescent individuals exhibited a bias toward elevated S-specific humoral immunity compared with N-specific immunity, in contrast to individuals who later passed away (Figure 4B;

Table 2. Demographics of the SARS-CoV-2 Cohort from Boston

Characteristics	Convalescent (N = 20)	Deceased (N = 20)
Female sex – no. (%)	6 (30)	6 (30)
Age (IQR)	56 (63–45)	78 (81.5–68)
Median days from onset of symptoms to sample collection (IQR)	9 (14.25–7.5)	8.5 (12–6)

Figure S4C). Thus, to ultimately capture the extent of S:N skewing across the groups, the number of features that had greater S than N responses were summed across convalescents and deceased individuals and compared within each cohort (Figures 4A and 4B). In both cohorts, a significant enrichment of S:N immunity was observed in convalescents (Figure 4C). Therefore, these findings suggest that a consistent overall shift in S:N immunity early in SARS-CoV-2 infection may have a protective role and aid in recovery from severe disease.

DISCUSSION

Cellular and humoral immune responses have been linked to protection against several coronaviruses (Li et al., 2006). Importantly, antibodies are pathogen-specific markers of exposure, serve as powerful biomarkers of disease activity, and often point to immunological mechanisms of protection able to guide therapeutic or vaccine development (Gunn and Alter, 2016). By deeply profiling the SARS-CoV-2 humoral immune response early in infection, here we defined a unique SARS-CoV-2-specific humoral signature associated with later disease outcomes. A combination of five SARS-CoV-2-specific antibody measurements was sufficient to distinguish individuals with different disease trajectories in a cohort from Seattle, including antibody measurements to S and N, with an overall enhanced S-centric response in individuals who recovered from infection. S-specific phagocytic and complement activity were enriched early in individuals who recovered from infection. This signature was confirmed in a second, larger SARS-CoV-2 infection cohort from Boston, where convalescent individuals exhibited a higher S:N ratio in their humoral immune response. These data point to early diverging humoral immune responses that may mark more effective immunity and suggest that functional antibodies directed against S might be beneficial for SARS-CoV-2 disease trajectory.

In SARS-CoV-1 and SARS-CoV-2 infection, N is highly immunogenic, with N-specific humoral immune responses arising concurrently with S-specific humoral immunity (Liu et al., 2020; Shi et al., 2004; Timani et al., 2004). However, immunization of hamsters with a vector expressing N offered no protection against SARS-CoV-2 challenge despite a strong anti-N response, whereas immunization with the same vector expressing S protected hamsters against challenge (Buchholz et al., 2004). It is estimated that 100 copies of S and 1,000 copies of N are incorporated into each virion (Bar-On et al., 2020), suggesting that 10-fold more N may be produced compared with S during infection to effectively generate viral progeny. Because of the high amounts of N, N-directed responses may be indicative of higher disease burden and increased antigen exposure. However, the similarity in viral loads between individuals who recovered and those who died does not support this hypothesis. Rather, the data point to compromised evolution of S immunity in individuals who later pass away. The potential beneficial role of S-targeted immunity in viral control is reinforced in new studies in non-human primates (NHPs), demonstrating elevated and robust functional humoral immune responses to S, rather than RBD and N, following primary infection that were associated with protection upon re-exposure to the virus (Chandrashekar et al., 2020).

It is well known that the timing of sampling may influence humoral profiles, where sampling time could result in comparison of immature versus mature immune responses. Despite the sampling differences in the group, comparable titers were observed across convalescents and individuals who ultimately passed away. Moreover, similar overall functional profiles were also observed, suggesting that the humoral immune responses were comparable in magnitude across the two groups. Additional analysis of the influence of sampling time on the spread of the antibody profiles in the PLSDA highlighted a minimal influence of time from symptoms on overall antibody profile variation, and the time of sampling exhibited a minimal predictive power in classifying individuals into convalescents or deceased. However, longitudinal analyses will be illuminating, providing further information regarding the evolving humoral immune response that tracks with protection from infection.

Emerging data point to higher mortality among the elderly and across genders (Hauser et al., 2020). Along these lines, individuals who passed away were, on average, older than those who convalesced. Age can have a profound effect on immune function, and although this study was not suited to explore the relationship between age, outcome, and humoral responses, future larger studies across age groups could provide insights into the differential susceptibility among the elderly. However, the effect of age, sex, and viral load illustrated a minimal influence of each of these variables on the overall variability of the humoral immune responses. Additionally, the individual predictive power of these demographic variables was lower than the predictive power of the model-selected antibody features (LV1).

Although S-specific antibodies able to recruit NK cell activity were expanded in individuals who passed away, pointing to a potentially negative influence of NK cells, coordination of NK cell and phagocytic activity was enriched among convalescents. These seemingly contradictory data point to the potential importance of synergy between innate immune effector functions. Although NK cells have been implicated in protection (Lu et al., 2016; Jegaskanda et al., 2016; van Erp et al., 2019) and pathology (Cong and Wei, 2019), it is possible that the evolution of antibodies able to harness the cytotoxic power of NK cells to eliminate infected or phagocytic cells may play a critical role in elimination and clearance of the infection. Interestingly, this coordination was associated with the synergistic evolution of a broader isotype- and subclass-specific response among convalescents. However, whether additional changes in antibody-Fc-glycosylation also contribute to this unique functionalization of antibody isotypes and subclasses, enabling coordination, remains unclear but could point to promising target immune profiles that may confer the greatest level of protection against the virus.

CellPress
OPEN ACCESS

Limitations of Study

There are a number of limitations in this study. First, because these samples were collected early during the COVID-19 pandemic in the United States, the Seattle study included a small number of participants, and the groups were not age- or sex-matched. Confounding factors such as timing of sampling, sex, and age are known to influence SARS-CoV-2 infection and disease trajectory. Although antibody profiles clearly segregated individuals who survived compared with those who did not survive, more limited variation in antibody profiles was observed across age, sex, viral load, and days from symptom onset. However, among the co-morbidities, age was the second major driver of variation in antibody profiles, pointing a potentially critical role of age-associated defects in Fc variation that may contribute to altered antiviral immunity to SARS-CoV-2 and beyond. The larger validation cohort from Boston identified a similar humoral signature that discriminated survivors from non-survivors, highlighting the conserved nature of this immunological signature independent of demographic characteristics. Although this study only attempted to understand the humoral disparities between convalescent and deceased individuals in a cohort of severely infected individuals, future studies may attempt to define humoral profiles able to further classify individuals across the clinical trajectory spectrum ranging from asymptomatic to severe disease.

Collectively, the data presented here argue for the evolution of distinct antigen-specific and functional humoral immune responses early in SARS-CoV-2 disease. Although further analysis of longitudinal cohorts may provide more mechanistic insights into the specific role of antibodies in control and clearance of infection, here we validated an early functional humoral immune signature that appears to predict disease progression across two distinct cohorts. Linked to emerging animal model experiments, the correlates defined here may provide key mechanistic insights to guide therapeutic and vaccine design efforts.

STAR★METHODS

Detailed methods are provided in the online version of this paper and include the following:

- KEY RESOURCES TABLE
- RESOURCE AVAILABILITY
  - Lead Contact
  - Material Availability
  - Data and Code Availability
- EXPERIMENTAL MODEL AND SUBJECT DETAILS
  - Sample set
  - Primary Immune Cells
  - Cell Lines
- METHOD DETAILS
  - Luminex
  - Functional profiling
  - Pseudovirus Neutralization Antibody Assay
- QUANTIFICATION AND STATISTICAL ANALYSIS
  - Classification of Convalescent and Deceased Groups
  - Correlation Networks
  - Sensitivity Analysis
  - Ratio Based Analyses

SUPPLEMENTAL INFORMATION

Supplemental Information can be found online at https://doi.org/10.1016/j.immuni.2020.07.020.

ACKNOWLEDGMENTS

We thank Bruce Walker, Nancy Zimmerman, Mark and Lisa Schwartz, an anonymous donor, and Terry and Susan Ragon for support. We would also like to thank Bing Chen for protein production efforts. We acknowledge support from the Ragon Institute of MGH, MIT and Harvard, the Massachusetts Consortium on Pathogen Readiness (MassCPR), the Bill and Melinda Gates Foundation (235730), the NIAID (U19 AI135995) and the Centers for Disease Control and Prevention (CK000490).

AUTHOR CONTRIBUTIONS

S.F., C.A., H.C., and G.A. designed the study. S.F., C.A., M.D.S., and J.B. performed the immunological assays. H.C. collected and selected the clinical specimens. T.Z., C.L., and D.L. performed all analyses. E.T.R. and R.C.C. participated in sample collection, study design, and interpretation of data. A.G.S., J.F., T.C., B.M.H., and Y.C. provided purified proteins. G.A. wrote the paper with all co-authors.

DECLARATION OF INTERESTS

G.A. is a founder of SeromYx Systems.

Received: May 22, 2020
Revised: June 29, 2020
Accepted: July 23, 2020
Published: July 30, 2020

REFERENCES

Ackerman, M.E., Das, J., Pittala, S., Broge, T., Linde, C., Suscovich, T.J., Brown, E.P., Bradley, T., Natarajan, H., Lin, S., et al. (2018). Route of immunization defines multiple mechanisms of vaccine-mediated protection against SIV. Nat. Med. *24*, 1590–1598.

Bar-On, Y.M., Flamholz, A., Phillips, R., and Milo, R. (2020). SARS-CoV-2 (COVID-19) by the numbers. eLife *9*, e57309.

Barouch, D.H., Alter, G., Broge, T., Linde, C., Ackerman, M.E., Brown, E.P., Borducchi, E.N., Smith, K.M., Nkolola, J.P., Liu, J., et al. (2015). Protective efficacy of adenovirus/protein vaccines against SIV challenges in rhesus monkeys. Science *349*, 320–324.

Bhatraju, P.K., Ghassemieh, B.J., Nichols, M., Kim, R., Jerome, K.R., Nalla, A.K., Greninger, A.L., Pipavath, S., Wurfel, M.M., Evans, L., et al. (2020). Covid-19 in Critically Ill Patients in the Seattle Region - Case Series. N. Engl. J. Med. *382*, 2012–2022.

Boudreau, C.M., Yu, W.H., Suscovich, T.J., Talbot, H.K., Edwards, K.M., and Alter, G. (2020). Selective induction of antibody effector functional responses using MF59-adjuvanted vaccination. J. Clin. Invest. *130*, 662–672.

Brown, E.P., Dowell, K.G., Boesch, A.W., Normandin, E., Mahan, A.E., Chu, T., Barouch, D.H., Bailey-Kellogg, C., Alter, G., and Ackerman, M.E. (2017). Multiplexed Fc array for evaluation of antigen-specific antibody effector profiles. J. Immunol. Methods *443*, 33–44.

Buchholz, U.J., Bukreyev, A., Yang, L., Lamirande, E.W., Murphy, B.R., Subbarao, K., and Collins, P.L. (2004). Contributions of the structural proteins of severe acute respiratory syndrome coronavirus to protective immunity. Proc. Natl. Acad. Sci. USA *101*, 9804–9809.

Chandrashekar, A., Liu, J., Martinot, A.J., McMahan, K., Mercado, N.B., Peter, L., Tostanoski, L.H., Yu, J., Maliga, Z., Nekorchuk, M., et al. (2020). SARS-CoV-2 infection protects against rechallenge in rhesus macaques. Science, eabc4776.

Chang, S.C., Wang, J.T., Huang, L.M., Chen, Y.C., Fang, C.T., Sheng, W.H., Wang, J.L., Yu, C.J., and Yang, P.C. (2005). Longitudinal analysis of Severe

Acute Respiratory Syndrome (SARS) coronavirus-specific antibody in SARS patients. Clin. Diagn. Lab. Immunol. *12*, 1455–1457.

Chen, G., Wu, D., Guo, W., Cao, Y., Huang, D., Wang, H., Wang, T., Zhang, X., Chen, H., Yu, H., et al. (2020). Clinical and immunological features of severe and moderate coronavirus disease 2019. J Clin Invest. *130*, 2620–2629.

Cobey, S., and Hensley, S.E. (2017). Immune history and influenza virus susceptibility. Curr. Opin. Virol. *22*, 105–111.

Cong, J., and Wei, H. (2019). Natural Killer Cells in the Lungs. Front. Immunol. *10*, 1416.

de Wit, E., van Doremalen, N., Falzarano, D., and Munster, V.J. (2016). SARS and MERS: recent insights into emerging coronaviruses. Nat. Rev. Microbiol. *14*, 523–534.

Fischinger, S., Fallon, J.K., Michell, A.R., Broge, T., Suscovich, T.J., Streeck, H., and Alter, G. (2019). A high-throughput, bead-based, antigen-specific assay to assess the ability of antibodies to induce complement activation. J. Immunol. Methods *473*, 112630.

Gunn, B.M., and Alter, G. (2016). Modulating Antibody Functionality in Infectious Disease and Vaccination. Trends Mol. Med. *22*, 969–982.

Guyon, I., and Elisseeff, A. (2003). An Introduction to Variable and Feature Selection. J. Mach. Learn. Res. *3*, 1157–1182.

Hauser, A., Counotte, M.J., Margossian, C.C., Konstantinoudis, G., Low, N., Althaus, C.L., and Riou, J. (2020). Estimation of SARS-CoV-2 Mortality during the Early Stages of an Epidemic: A Modelling Study in Hubei, China and Northern Italy. medRxiv. https://doi.org/10.1101/2020.03.04.20031104.

He, Y., Li, J., and Jiang, S. (2006). A single amino acid substitution (R441A) in the receptor-binding domain of SARS coronavirus spike protein disrupts the antigenic structure and binding activity. Biochem. Biophys. Res. Commun. *344*, 106–113.

Holshue, M.L., DeBolt, C., Lindquist, S., Lofy, K.H., Wiesman, J., Bruce, H., Spitters, C., Ericson, K., Wilkerson, S., Tural, A., et al.; Washington State 2019-nCoV Case Investigation Team (2020). First Case of 2019 Novel Coronavirus in the United States. N. Engl. J. Med. *382*, 929–936.

Jegaskanda, S., Luke, C., Hickman, H.D., Sangster, M.Y., Wieland-Alter, W.F., Mcbride, J.M., Yewdell, J.W., Wright, P.F., Treanor, J., Rosenberger, C.M., and Subbarao, K. (2016). Generation and Protective Ability of Influenza Virus-Specific Antibody-Dependent Cellular Cytotoxicity in Humans Elicited by Vaccination, Natural Infection, and Experimental Challenge. J. Infect. Dis. *214*, 945–952.

Lau, K.S., Juchheim, A.M., Cavaliere, K.R., Philips, S.R., Lauffenburger, D.A., and Haigis, K.M. (2011). In vivo systems analysis identifies spatial and temporal aspects of the modulation of TNF-α-induced apoptosis and proliferation by MAPKs. Sci. Signal. *4*, ra16.

Li, T., Xie, J., He, Y., Fan, H., Baril, L., Qiu, Z., Han, Y., Xu, W., Zhang, W., You, H., et al. (2006). Long-term persistence of robust antibody and cytotoxic T cell responses in recovered patients infected with SARS coronavirus. PLoS ONE *1*, e24.

Li, R., Pei, S., Chen, B., Song, Y., Zhang, T., Yang, W., and Shaman, J. (2020). Substantial undocumented infection facilitates the rapid dissemination of novel coronavirus (SARS-CoV2). Science *368*, 489–493.

Liu, W., Liu, L., Kou, G., Zheng, Y., Ding, Y., Ni, W., Wang, Q., Tan, L., Wu, W., Tang, S., et al. (2020). Evaluation of Nucleocapsid and Spike Protein-based ELISAs for detecting antibodies against SARS-CoV-2. J. Clin. Microbiol. *58*, e00461-20.

Lu, L.L., Chung, A.W., Rosebrock, T.R., Ghebremichael, M., Yu, W.H., Grace, P.S., Schoen, M.K., Tafesse, F., Martin, C., Leung, V., et al. (2016). A Functional Role for Antibodies in Tuberculosis. Cell *167*, 433–443.e14.

Ojala, M., and Garriga, G. (2010). Permutation tests for studying classifier performance. J. Mach. Learn. Res. *11*, 1833–1863.

Pedregosa, F., Varoquaux, G., Gramfort, A., Michel, V., Thirion, B., Grisel, O., Blondel, M., et al. (2011). Scikit-Learn: Machine Learning in Python. J. Mach. Learn. Res. *12*, 2825–2830.

Pittala, S., Bagley, K., Schwartz, J.A., Brown, E.P., Weiner, J.A., Prado, I.J., Zhang, W., Xu, R., Ota-Setlik, A., Pal, R., et al. (2019). Antibody Fab-Fc properties outperform titer in predictive models of SIV vaccine-induced protection. Mol. Syst. Biol. *15*, e8747.

Quinlan, B.D., Mou, H., Zhang, L., Guo, Y., He, W., Ojha, A., Parcells, M.S., Luo, G., Li, W., Zhong, G., et al. (2020). The SARS-CoV-2 receptor-binding domain elicits a potent neutralizing response without antibody-dependent enhancement. bioRxiv. https://doi.org/10.1101/2020.04.10.036418.

Saphire, E.O., Schendel, S.L., Gunn, B.M., Milligan, J.C., and Alter, G. (2018). Antibody-mediated protection against Ebola virus. Nat. Immunol. *19*, 1169–1178.

Shi, Y., Wan, Z., Li, L., Li, P., Li, C., Ma, Q., and Cao, C. (2004). Antibody responses against SARS-coronavirus and its nucleocaspid in SARS patients. J. Clin. Virol. *31*, 66–68.

ter Meulen, J., van den Brink, E.N., Poon, L.L., Marissen, W.E., Leung, C.S., Cox, F., Cheung, C.Y., Bakker, A.Q., Bogaards, J.A., van Deventer, E., et al. (2006). Human monoclonal antibody combination against SARS coronavirus: synergy and coverage of escape mutants. PLoS Med. *3*, e237.

Timani, K.A., Ye, L., Ye, L., Zhu, Y., Wu, Z., and Gong, Z. (2004). Cloning, sequencing, expression, and purification of SARS-associated coronavirus nucleocapsid protein for serodiagnosis of SARS. J. Clin. Virol. *30*, 309–312.

Tomaras, G.D., and Haynes, B.F. (2009). HIV-1-specific antibody responses during acute and chronic HIV-1 infection. Curr. Opin. HIV AIDS *4*, 373–379.

van Erp, E.A., Luytjes, W., Ferwerda, G., and van Kasteren, P.B. (2019). Fc-Mediated Antibody Effector Functions During Respiratory Syncytial Virus Infection and Disease. Front. Immunol. *10*, 548.

Wang, X., Guo, X., Xin, Q., Pan, Y., Li, J., Chu, Y., Feng, Y., and Wang, Q. (2020). Neutralizing Antibodies Responses to SARS-CoV-2 in COVID-19 Inpatients and Convalescent Patients. Clin. Infect. Dis. Published online June 4, 2020. https://doi.org/10.1093/cid/ciaa721.

World Health Organization (2020). Coronavirus disease (COVID-19) Situtation Report 122. https://www.who.int/docs/default-source/coronaviruse/situation-reports/20200211-sitrep-22-ncov.pdf?sfvrsn=fb6d49b1_2.

Wu, F., Wang, A., Liu, M., Wang, Q., Chen, J., Xia, S., Ling, Y., Zhang, Y., Xun, J., Lu, L., et al. (2020a). Neutralizing antibody responses to SARS-CoV-2 in a COVID-19 recovered patient cohort and their implications. medRxiv. https://doi.org/10.1101/2020.03.30.20047365.

Wu, F., Zhao, S., Yu, B., Chen, Y.M., Wang, W., Song, Z.G., Hu, Y., Tao, Z.W., Tian, J.H., Pei, Y.Y., et al. (2020b). A new coronavirus associated with human respiratory disease in China. Nature *579*, 265–269.

Yang, Z.Y., Kong, W.P., Huang, Y., Roberts, A., Murphy, B.R., Subbarao, K., and Nabel, G.J. (2004). A DNA vaccine induces SARS coronavirus neutralization and protective immunity in mice. Nature *428*, 561–564.

Yasui, F., Kohara, M., Kitabatake, M., Nishiwaki, T., Fujii, H., Tateno, C., Yoneda, M., Morita, K., Matsushima, K., Koyasu, S., and Kai, C. (2014). Phagocytic cells contribute to the antibody-mediated elimination of pulmonary-infected SARS coronavirus. Virology *454-455*, 157–168.

Zhao, J., Perera, R.A., Kayali, G., Meyerholz, D., Perlman, S., and Peiris, M. (2015). Passive immunotherapy with dromedary immune serum in an experimental animal model for Middle East respiratory syndrome coronavirus infection. J. Virol. *89*, 6117–6120.

Zhao, Y., Wang, C., Qiu, B., Li, C., Wang, H., Jin, H., Gai, W., Zheng, X., Wang, T., Sun, W., et al. (2017). Passive immunotherapy for Middle East Respiratory Syndrome coronavirus infection with equine immunoglobulin or immunoglobulin fragments in a mouse model. Antiviral Res. *137*, 125–130.

Zhu, N., Zhang, D., Wang, W., Li, X., Yang, B., Song, J., Zhao, X., Huang, B., Shi, W., Lu, R., et al.; China Novel Coronavirus Investigating and Research Team (2020). A Novel Coronavirus from Patients with Pneumonia in China, 2019. N. Engl. J. Med. *382*, 727–733.

CellPress
OPEN ACCESS

STAR★METHODS

KEY RESOURCES TABLE

REAGENT or RESOURCE	SOURCE	IDENTIFIER
**Antibodies**		
anti-CD66b-Pacific blue	BioLegend	CAT#: 305112
APC-Cy7 Mouse Anti-Human CD16	BD Biosciences	CAT#557758; RRID:AB_396853
CD56 PE-Cy7 Mouse Anti-Human CD56	BD Biosciences	CAT#557747
PE MIP-1b Mouse anti-Human	BD Biosciences	CAT#550078; RRID:AB_393549
Pacific Blue Mouse Anti-Human CD3	BD Biosciences	CAT#558117; RRID:AB_1595437
Anti-Human IgG (Fc specific), highly cross adsorbed-Peroxidase antibody produced in goat	Sigma-Aldrich	CAT#: SAB3701283-1MG
FITC Goat IgG anti-C3	MP Biomedicals	CAT#: 855385
Mouse Anti-Human IgG1-Fc PE	Southern Biotech	CAT # 9054-09
Mouse Anti-Human IgG2-Fc PE	Southern Biotech	CAT # 9060-09
Mouse Anti-Human IgG3-Hinge PE	Southern Biotech	CAT # 9210-09
Mouse Anti-Human IgG4-Fc PE	Southern Biotech	CAT # 9200-09
Mouse Anti-Human IgA1-Fc PE	Southern Biotech	CAT # 9130-09
Mouse Anti-Human IgM-Fc PE	Southern Biotech	CAT # 9020-09
BV605 Mouse Anti-Human CD11b	BD Biosciences	CAT# 562721
**Bacterial and Virus Strains**		
SARS-CoV-2-S pseudovirus with a luciferase reporter	This paper	
**Chemicals, Peptides, and Recombinant Proteins**		
SARS-CoV-2 S	Obtained from the lab of Dr. Eric Fischer	
SARS-CoV-2 RBD	Obtained from the lab of Dr. Aaron Schmidt	
SARS-CoV-2 N	Aalto Bio Reagents	CAT # CK 6404-b
Human Fc receptors	Produced at the Duke HumanVaccine Institute, {Boesch, 2014 #15}	
Streptavidin-R-Phycoerythrin	Prozyme	CAT#:PJ31S
FIX&Perm Cell Permeabilization Kit	Life Tech	CAT#: GAS001S100, GAS002S100
Brefeldin A	Sigma Aldrich	CAT#: B7651
GolgiStop	BD Biosciences	CAT#: 554724
Luciferase Assay Reagent	Promega	CAT#: E1483
**Critical Commercial Assays**		
BirA-500: BirA biotin-protein ligase standardreaction kit	Avidity	CAT#: BirA500
RosetteSep Human NK Cell Enrichment Cocktail	Stem Cell Technologies	CAT#: 15065
Steady-Glo Luciferase Assay	Promega	CAT#: E2510
**Experimental Models: Cell Lines**		
THP-1 Cells	ATCC	CAT#: TIB-202; RRID: CVCL_0006
**Recombinant DNA**		
psPAX2	AIDS Reagent	CAT#11348
pLenti-CMV Puro-Luc	Addgene	CAT#17447
pcDNA3.1-SARS CoV-2.SΔCT	This paper	
pcDNA3.1(-)-hACE2	Addgene	CAT# 1786

(*Continued on next page*)

Continued		
REAGENT or RESOURCE	SOURCE	IDENTIFIER
Software and Algorithms		
GraphPad Prism	GraphPad	https://www.graphpad.com/scientificsoftware/prism/
Intellicyt ForeCyt Software	Sartorious	https://intellicyt.com/products/software/
Python programming language	Version 3.6.8	https://www.python.org/
Other		
FluoSpheres NeutrAvidin-Labeled Microspheres, 1.0 μm, yellow-green fluorescent (505/515), 1% solids	Invitrogen	CAT#: F8776
FluoSpheres NeutrAvidin-Labeled Microspheres, 1.0 μm, red fluorescent (505/515), 1% solids	Invitrogen	CAT#: F8775
MagPlex microspheres	Luminex corporation	CAT#: MC12001-01, MCI12040-01, MCI10077-01

RESOURCE AVAILABILITY

Lead Contact

Further information and requests for resources and reagents should be directed to and will be fulfilled by the Lead Contact, Galit Alter (galter@partners.org).

Material Availability

This study did not generate new unique reagents.

Data and Code Availability

The dataset generated during and/or analyzed during the current study have been made available in the supplemental material.

EXPERIMENTAL MODEL AND SUBJECT DETAILS

Sample set

Plasma samples from 22 SARS-CoV-2 patients from Seattle were profiled for anti-SARS-CoV-2 antibody responses (Table 1). Patients who tested positive for SARS-CoV-2 by real-time reverse-transcriptase–polymerase-chain-reaction (RT-PCR) of a nasopharyngeal swab were enrolled in the study upon hospital admission, and samples after admission were included in this study (Figure S1). All enrolled participants gave written, informed consent. The enrolled hospitalized 22 individuals were monitored over the course of their stay, and final outcomes were reported. 12 individuals convalesced and were healthy enough to be discharged, whereas 10 individuals died. Demographic information including age, race, and interventions are summarized across the two groups (Table 1; Data S1).

As a validation cohort, a cohort of 40 individuals from MGH in Boston were enrolled, all participants tested positive for SARS-CoV-2 by RT-PCR and they were monitored over their hospital stay. Samples at time of hospitalization were included in this study. Outcomes were reported as deceased or discharged. Demographics and clinical data for the validation cohort are summarized in Table 2.

All experimental data was performed in two technical and two biological (for primary cell assays) replicates and the average value was used throughout the study. This study was approved by the University of Washington Human Subjects Division Institutional Review Board.

Primary Immune Cells

Primary immune cells were isolated from fresh peripheral blood from healthy human volunteers collected by the MGH Blood bank or the Ragon institute. The study was approved by the MGH Institutional Review Board. All subjects were over 18 years of age and provided informed consent. All samples were completely de-identified prior to use. Human NK cells and neutrophils isolated from fresh peripheral blood were cultured in RPMI supplemented with with 10% fetal bovine serum, L-glutamine, penicillin/streptomycin and maintained at 37°C, 5% CO_2.

Cell Lines

THP-1 cells (ATCC) were grown at 37°C, 5% CO_2 in RPMI supplemented with 10% fetal bovine serum, L-glutamine, penicillin/streptomycin and 0.01% b-mercaptoethanol.

METHOD DETAILS

Luminex

Antigen-specific antibody subclass, isotype, sialic acid, galactose and Fcγ-receptor (FcγR) binding levels were assessed using a 384-well based customized multiplexed Luminex assay, as previously described (Brown et al., 2017) Relative antibody concentration was measured against a panel of SARS-CoV-2 antigens (Data S1). SARS-CoV-2 RBD (kindly provided by Aaron Schmidt), SARS-CoV-2 nucleocapsid (N) protein (Aalto Bio Reagents), and SARS-CoV-2 spike protein (S) (kindly provided by Bing Chen) were used to profile the SARS-CoV-2-specific humoral immune response. Briefly, antigens were coupled by covalent NHS-ester linkages via EDC and NHS (Thermo Scientific) to fluorescent carboxyl- modified microspheres (Luminex). Antigen-coupled microspheres were then washed with an automated plate washer (Tecan) and incubated with plasma samples at an appropriate sample dilution (1:500 for IgG1 and all Fcγ- receptors, and 1:100 for all other readouts). Detection of antigen-specific antibody titers occurred using a PE-coupled detection antibody for each subclass and isotype (IgG, IgG1, IgG2, IgG3, IgG4, IgA1 and IgM, Southern Biotech), and Fc©-receptors were fluorescently labeled with PE before addition to immune complexes (FcγR2A, 2B, 3A, Duke Protein Production facility). For detection of sialic acid and galactose, fluorescein-labeled plant-based lectin detects, SNA and RCA (Vectorlabs) were added as detection reagents at a 1:100 (SNA) and 1:500 dilution (RCA). Plasma samples were acquired via flow cytometry, using an iQue (Intellicyt) and S-Lab robot (PAA). Analysis was done using ForeCyt software by gating on fluorescent bead regions and PE median fluorescent intensity (MFI) is reported as readout for antigen-specific antibody titers.

Functional profiling

For the functional analysis of plasma samples, bead-based assays were used to quantify antibody-dependent cellular phagocytosis (ADCP), antibody-dependent neutrophil phagocytosis (ADNP) and antibody-dependent complement deposition (ADCD), as previously described (Fischinger et al., 2019; Data S1). Fluorescent streptavidin beads (Thermo Fisher) were coupled to biotinylated antigen SARS-CoV-2 RBD, N and S and incubated with diluted plasma (ADCP and ADNP 1:100, ADCD 1:10). For ADCP, THP-1 cells were added to the immune complexes and incubated for 16h at 37°C. For ADNP, primary neutrophils were isolated via negative selection (Stemcell) from whole blood. After 1h incubation at 37°C, neutrophils were stained with an anti-CD66b PacBlue detection antibody (Biolegend). For the ADCD assay, lyophilized guinea pig complement (Cedarlane) was resuspended according to manufacturer's instructions and diluted in gelatin veronal buffer with calcium and magnesium (Boston BioProducts). Post incubation, C3 was detected with Fluorescein-Conjugated Goat IgG Fraction to Guinea Pig Complement C3 (Mpbio).

For detection of antibody-dependent NK cell activity, an ELISA-based approach was used, as described (Boudreau et al., 2020). Briefly, plates were coated with 2 μg/mL of antigen (as mentioned above) and samples were added at a 1:50 dilution and incubated for 2h at 37°C. NK cells were isolated the day prior via RosetteSep (Stem Cell Technologies) from healthy buffy coats and rested overnight in 1 ng/ml IL-15 (Stemcell). NK cells were incubated with immune complexes for 5h at 37°C with a staining cocktail containing CD107a PE-Cy5 (BD), Golgi stop (BD) and Brefeldin A (BFA, Sigma Aldrich). Post NK cell incubation, cells were fixed (Perm A, Life Tech) and stained for surface markers with anti-CD16 APC-Cy7 (BD), anti-CD56 PE-Cy7 (BD) and anti-CD3 PacBlue (BD) while fixing. Post permeabilization with Perm B (Life Tech) and anti-MIP-1β PE (BD) antibodies were used for intracellular staining. All assays were acquired via flow cytometry with an iQue (Intellicyt) and an S-Lab robot (PAA). For ADCP, events were gated on bead-positive cells, whereas neutrophils were defined as CD66b positive followed by gating on bead-positive neutrophils. A phagocytosis score was calculated for ADCP and ADNP as (percentage of bead-positive cells) x (MFI of bead-positive cells) divided by 10000. ADCD was reported as MFI of C3 deposition. NK cells were defined as CD3-, CD16+ and CD56+. Data were reported as percentage of cells positive for CD107a or MIP-1β.

Pseudovirus Neutralization Antibody Assay

The 2019-nCoV pseudoviruses expressing a luciferase reporter gene were generated as described previously (Data S1) (Yang et al., 2004). Briefly, the packaging construct psPAX2 (Cat# 11348, AIDS Reagent), luciferase reporter plasmid pLenti-CMV Puro-Luc (Cat# 17447, Addgene) and Spike protein expressing pcDNA3.1-SARS CoV-2.SΔCT were co-transfected into HEK293T cells at ratio of 1:1:0.5 by Calcium phosphate transfection method. The supernatants containing the pseudotype viruses were collected 48 hours post-transfection and filtered by 0.45-μm filter. The viruses were stored at −80°C freezer till use. To determine the neutralization activity of the antisera from vaccinated animals, HEK293T cells were first transfected with pcDNA3.1(-)-hACE2 (Cat# 1786, Addgene). 12 hours post transfection; the HEK293T/hACE2 cells were seeded at 96-well tissue culture plate at density of 2.00E+04 cells/well overnight. Heat (56°C, 30 min) inactivated antisera were twofold serial diluted and mixed with 50μl of pseudoviruses. The mixture was incubated at 37°C incubator for 1 hour before adding into HEK293T/hACE2 cells in 96-well plates. Six hours after infection, the cell culture medium was replenished with fresh DMEM (supplemented with 2% FBS). Forty-eight hours after infection, cells were lysed in Steady-Glo Luciferase Assay (Promega). A standard quantity of cell lysate was used in a luciferase assay with luciferase assay reagent (Promega) according to the manufacturer's protocol.

QUANTIFICATION AND STATISTICAL ANALYSIS

All analyses were performed using python version 3.6.8 with statistical and machine learning packages (Pedregosa et al., 2011). Networks were visualized in Cytoscape. Raw data are available in supplementary information.

Classification of Convalescent and Deceased Groups

The classification models were trained to distinguish convalescent and deceased groups with a minimal set of features, to avoid overfitting. PBS controls was subtracted from all features, Fc array features were log transformed, and all data was scaled and centered. Antibody features including sex and interventions (Table 1) were included the selection process, and covariates were binarized and scaled and center prior to analysis.

The models were built using a backward feature elimination for selection and then classified using the minimal set of features which maximize accuracy (Guyon and Elisseeff, 2003; Pittala et al., 2019). Models were trained and tested in a fivefold cross-validation framework using random stratified sampling to ensure that both groups are represented in each group. Within each fold, samples were further subdivided into four sets for each iterative fold-specific elimination. A partial least-squares discriminant analysis (PLS) classifier was then trained using the fold-specific selected features to predict the test set. Multiple iterations of fold specific feature selections were performed to obtain a single model. This process was repeated over twenty replicates and convergent correlates were observed (Ackerman et al., 2018).

Performance and robustness of the model was contrasted with negative control models constructed from permuted data and randomly selected size-matched features, with multiple iterations of fivefold cross-validation used to generate classification accuracies. These control models were generated 100 times. The permuted control was generated in the same process as above shuffling labels randomly for each repetition. Size-matched features were chosen at random for each cross-validation step within each repetition. Predicted and true outcomes were compared to determine accuracy. Robustness was defined as the effect size of the distributions (Cliff's Δ), and the exact P values of the tail probabilities of the true distributions within the control distributions. Reported are the median p values across twenty independent cross-validation replicates (Ojala and Garriga, 2010).

Correlation Networks

Correlation networks were constructed to visualize the additional humoral immune features that were significantly linked to the selected minimal biomarkers, to provide enhanced insights into the biological mechanisms by which antibodies may provide protection following infection. In brief, antibody features that were significantly correlated with a Holms-Bonferroni correction to the final selected PLS model selected-features were defined as co-correlates. Significant spearman correlations above a threshold of $|r| > 0.5$ were visualized within the networks.

Sensitivity Analysis

Using the selected features from the original model a new PLSDA model was trained excluding a single outlier at a time in a fivefold cross validation framework. This process was repeated three times, each time generating a unique ROC curve as the top 3 individual outliers were removed. Using these cross validated ROC curves the mean performance and variation were assessed and are summarized as area under curve.

Ratio Based Analyses

In order to evaluate S versus N ratios, first ratios for each feature were defined separately by simply dividing S-responses over N-responses for every given feature. S:N ratios were visualized by log2 transformation for ease of interpretation. Differences across convalescents and deceased were then tested with a one-sided Mann-Whitney U test and a Holm-Bonferroni multiple hypothesis correction criterion.

In order to address whether the overall S-response was enriched over N-responses in the convalescents across all features tested, all data was background corrected and z-score normalized. Then the number of S-features which were greater than their N-counterparts across every feature were summed. This analysis yielded a distribution of individual S greater than N scores for each group and statistical differences were assessed using a one-sided Mann-Whitney U test.

Immunity, Volume *53*

Supplemental Information

Distinct Early Serological Signatures

Track with SARS-CoV-2 Survival

Caroline Atyeo, Stephanie Fischinger, Tomer Zohar, Matthew D. Slein, John Burke, Carolin Loos, Denise J. McCulloch, Kira L. Newman, Caitlin Wolf, Jingyou Yu, Kiel Shuey, Jared Feldman, Blake Marie Hauser, Tim Caradonna, Aaron G. Schmidt, Todd J. Suscovich, Caitlyn Linde, Yongfei Cai, Dan Barouch, Edward T. Ryan, Richelle C. Charles, Douglas Lauffenburger, Helen Chu, and Galit Alter

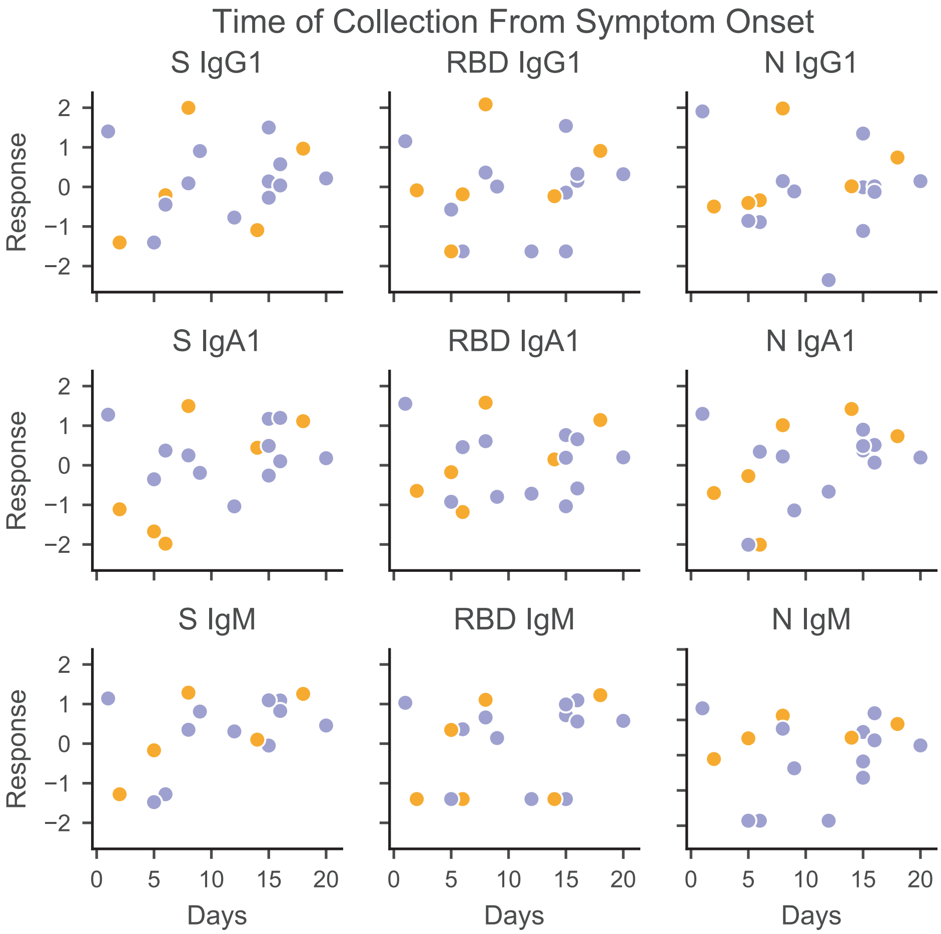

Figure S1: Individual antibody dynamics over time, related to Figure 1. The dot plots show the relationship of antibody titers against IgG1, IgA1, and IgM and the estimated time of collection from onset of symptoms across SARS-CoV-2 antigens.

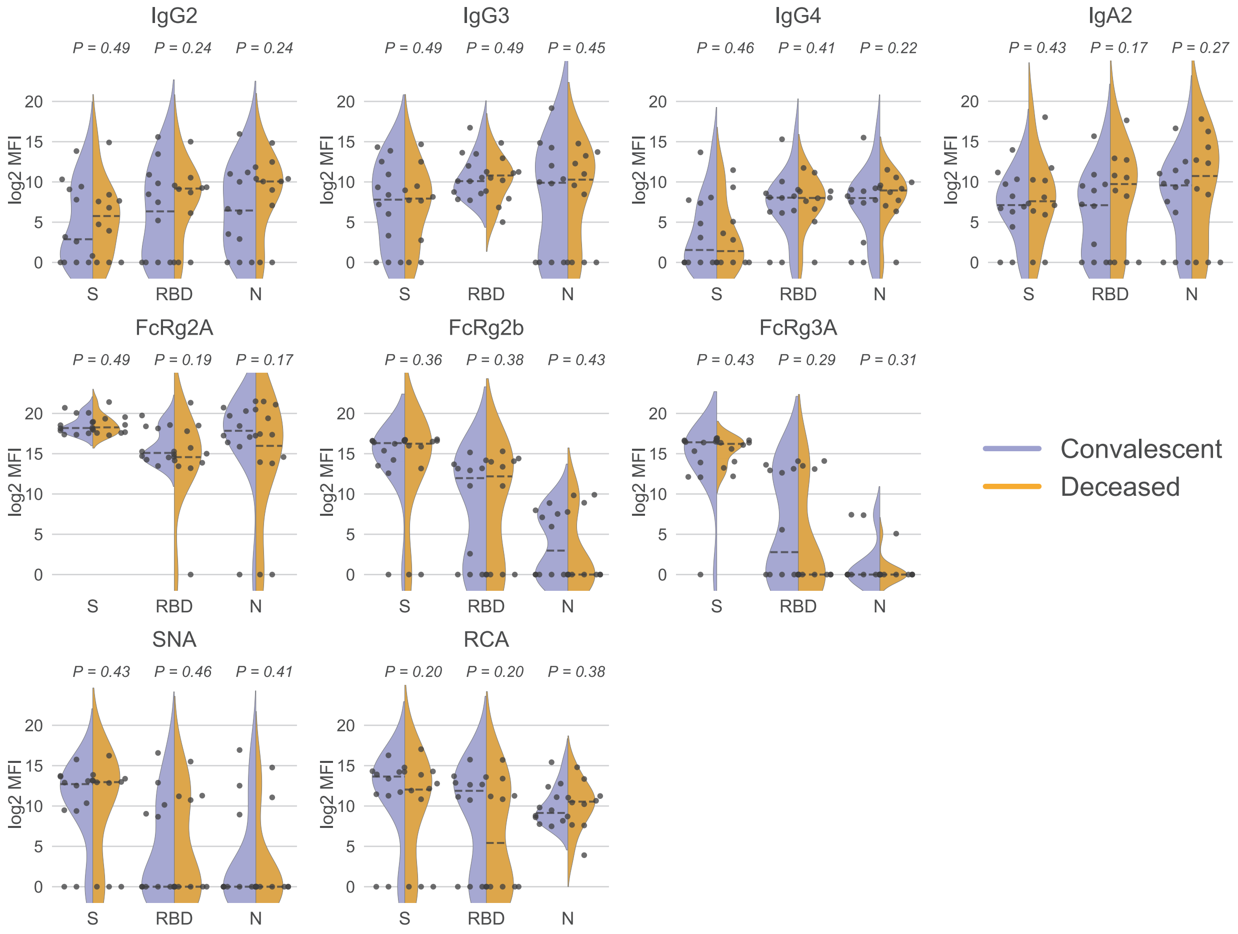

Figure S2: Individual antibody distributions, related to Figure 1. The split violin plots show the distribution of subclass, isotype, and Fc-receptor binding profiles across convalescent (purple) and deceased (orange) individuals. A Mann-Whitney U test was used to calculate p values. No significance was detected after a Holm-Bonferroni correction for multiple hypothesis testing.

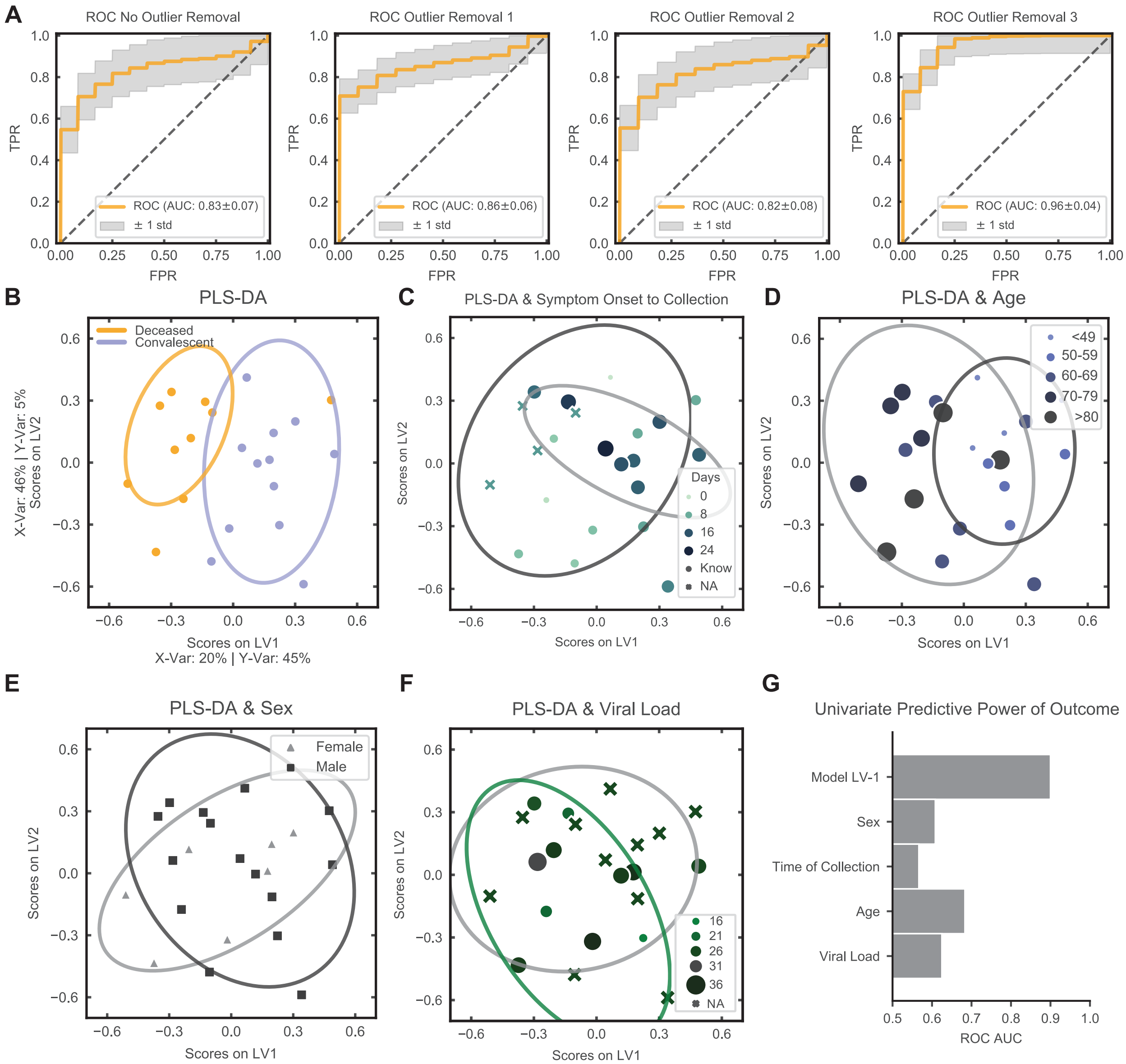

Figure S3: Sensitivity analysis, model performance, and potential influence of confounders, related to Figure 3. (**A**) Using the selected features from the original model, outliers were removed one at a time and ROC curves based on scores generated multiple times in a 5-fold cross validation framework were generated. In orange is the average performance and the grey shading represents one standard deviation. The gray dashed line represents the threshold of a random process. The AUC of the ROC curves and uncertainties are listed at the bottom of each graph. (FPR: false positive rate, TPR: true positive rate). (**B**) The original PLSDA scores plot is plotted based on the final antibody feature selection. (**C**) The same scores plot was recolored to highlight the distribution of the individuals based on days of symptom onset. The size and intensity of the dots from bright green to dark blue correspond to shorter-to-longer times, and x markers represent samples for which information was not available. (**D**) The same original scores plot was colored to reflect differences in ages across the subjects, with increased dots size and color (light blue to dark gray) showing increasing age. (**E**) The original scores plot was recolored to show the influence of sex on distributions, with males shown as dark squares and females as light gray triangles. (**F**) The original scores plot was recolored to show viral loads, with larger dots and increasing darkness (green to black) highlighting higher viral loads. (**G**) The bar graph shows the predictive power (as ROC AUC)of the original model latent variable 1 (LV1) compared to the univariate predictive power of individual demographic confounders, including sex, time to collection, age, and viral loads.

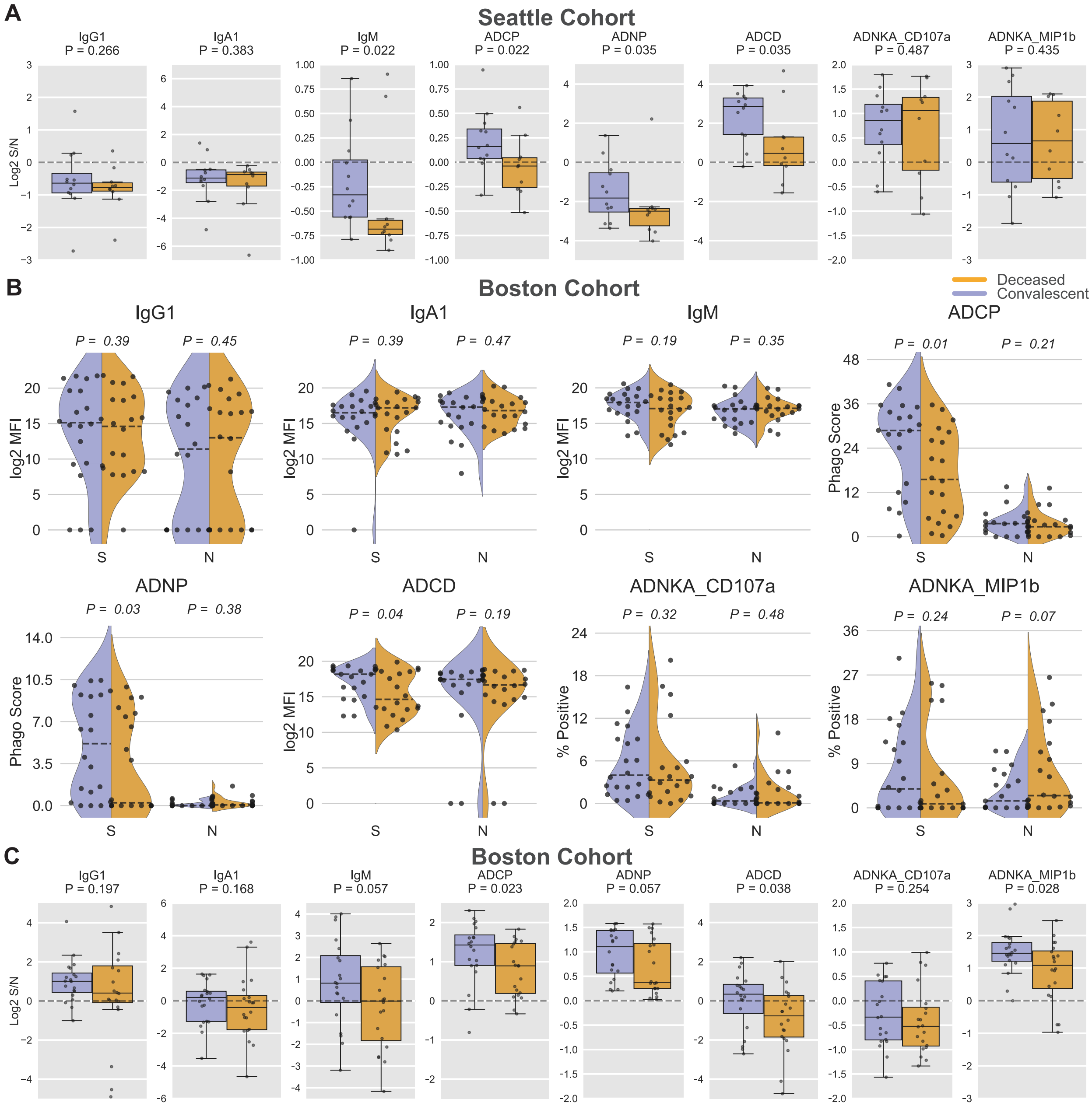

Figure S4 Univariate Spike:Nucleocapsid ratios across the Seattle/Discovery and Boston/Validation cohorts, related to Figure 4. The whisker plots show the log2 ratio of Spike to Nucleocapsid (S:N) in the (**A**) Seattle/Discovery cohort for IgG1, IgA1, IgM, antibody dependent cellular phagocytosis (ADCP), antibody dependent neutrophil phagocytosis (ADNP), antibody dependent complement deposition (ADCD), and antibody dependent NK cell activation (ADNKA) by degranulation (CD107a) and chemokine secretion (MIP1b) (from left to right). (**B**) The violin plots highlight the distributions of antibody features captured in the Boston/Validation cohort of IgG1, IgA1, IgM titers, ADCD from left to right on the top line; as well as ADCP, ADNP, and ADNKA by degranulation CD107a or MIP1b, from left to right on the bottom row. Responses are shown for Spike (S) and Nucleocapsid (N) for each read out for the convalescents (blue) and the deceased (yellow). The dashed line represents the median of the distribution. (**C**) The whisker plots show the log2 ratio of Spike to Nucleocapsid (S:N) in the Boston/Validation cohort with the same features as in (A). For both A and C the hatched line represents the point where ratios are enriched in S over N and differences were tested with a one-sided Mann-Whitney U test.
